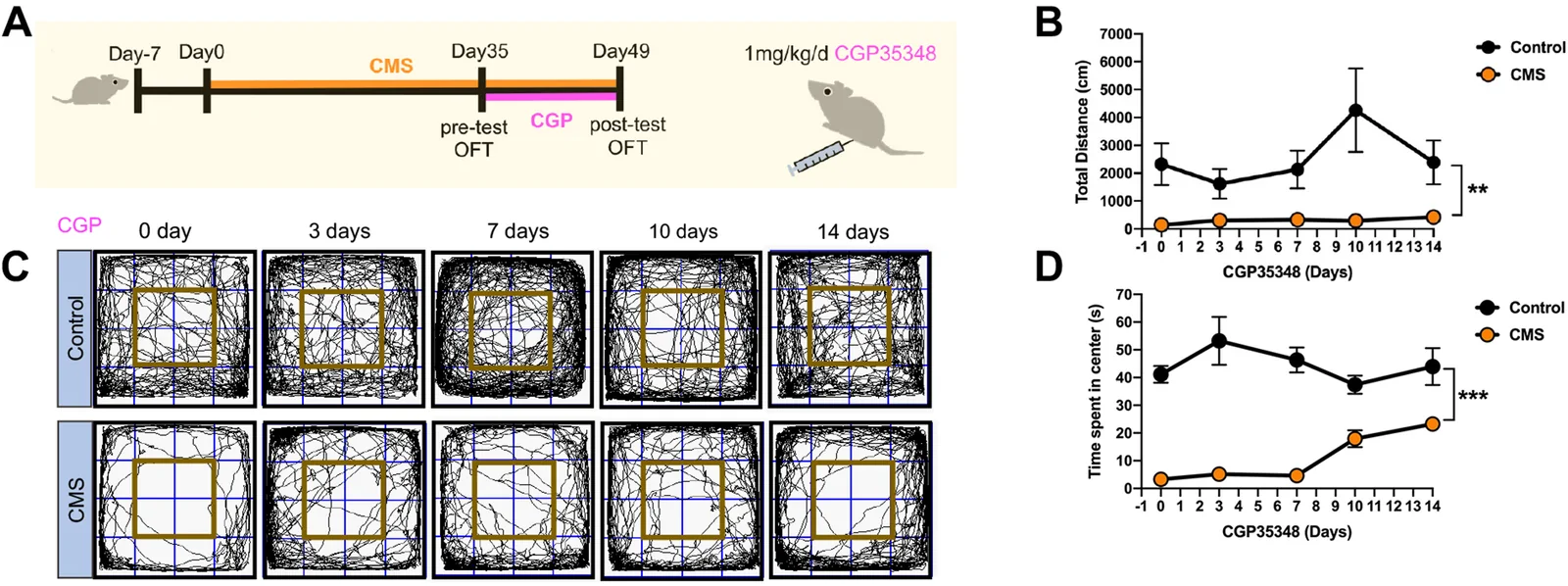# NAcLat-DBS reduces GABA_B_ expression of dopamine neurons to correct depressive symptoms in CMS mice

**DOI:** 10.1016/j.neurot.2026.e01039

**Published:** 2026-08-07

**Authors:** Jia-Ni Jing, Chao Yuan, Xia Li, Hao Fan

**Affiliations:** Department of Cognition and Brain Science, Beijing Institute of Basic Medical Sciences, Beijing, 100850, China

**Keywords:** Deep brain stimulation (DBS), Dopamine neurons, Ventral tegmental area (VTA), GABA_B_ receptor (GABA_B_), Chronic mild stress (CMS)

## Abstract

Hypodopaminergic activity within the mesolimbic circuitry is recognized as a typical characteristic in severe and refractory depression. Deep brain stimulation (DBS) of the nucleus accumbens (NAc) has shown therapeutic potential for treatment-resistant depression (TRD), yet its underlying molecular mechanism remains incompletely defined. We used chronic mild stress (CMS) combining fiber photometry recording, pharmacological interventions and genetic manipulations in freely moving mice to examine the variation of dopamine (DA) activity in the ventral tegmental area (VTA) and the potential roles of GABA_B_ receptor (GABA_B_) in mediating the effects of lateral nucleus accumbens shell (NAcLat)-targeted DBS. We performed in vivo fiber photometry recordings to track glutamatergic inputs onto the VTA after NAcLat-DBS. Immunofluorescence and in situ hybridization techniques were used to unravel GABA_B1_ expression of the DA and GABA neurons in the VTA. The results have determined that long-term NAcLat-DBS decreases DA GABA_B1_ expression and alleviates depressive-like behaviors in CMS mice. Importantly, selective overexpression of DA GABA_B1_ in VTA-NAcLat pathway reversed the behavioral benefits of DBS. This reduced DA GABA_B1_ expression was necessary for the antidepressant effects of DBS. Further, pharmacological blockade of GABA_B_ by CGP reproduced the antidepressant effects of DBS in CMS mice. Mechanistically, the enhanced excitation of glutamatergic input in the VTA involved in correcting dopaminergic activity and potentially involved in GABA_B_ expression alteration during NAcLat-DBS. These findings identify a previously unrecognized role of GABA_B_ in mediating the antidepressant outcome of NAcLat-DBS and suggest that modulation of the DA GABA_B1_ in VTA-NAcLat pathway could represent a pharmacological target for treatment-resistant depression.

## Graphical abstract

Jing et al. demonstrated that NAcLat-DBS reduced the GABA_B1_ expression of dopamine neurons in the VTA. Pharmacological blockade of GABA_B_ by CGP reproduced the antidepressant-like effects of DBS in CMS mice. Conversely, selective overexpression of DA GABA_B1_ in the VTA-NAcLat pathway reversed the behavioral benefits of DBS. Mechanistically, DBS decreased the surface expression of GABA_B_, and the enhanced excitation of glutamatergic input potentially involved in the GABA_B_ expression alteration during NAcLat-DBS. The reduced membrane GABA_B_ expression is necessary for the antidepressant effect of DBS. These findings suggest that modulation of DA GABA_B1_ in VTA-NAcLat pathway could represent a pharmacological target for treatment-resistant depression.

**Figure:**
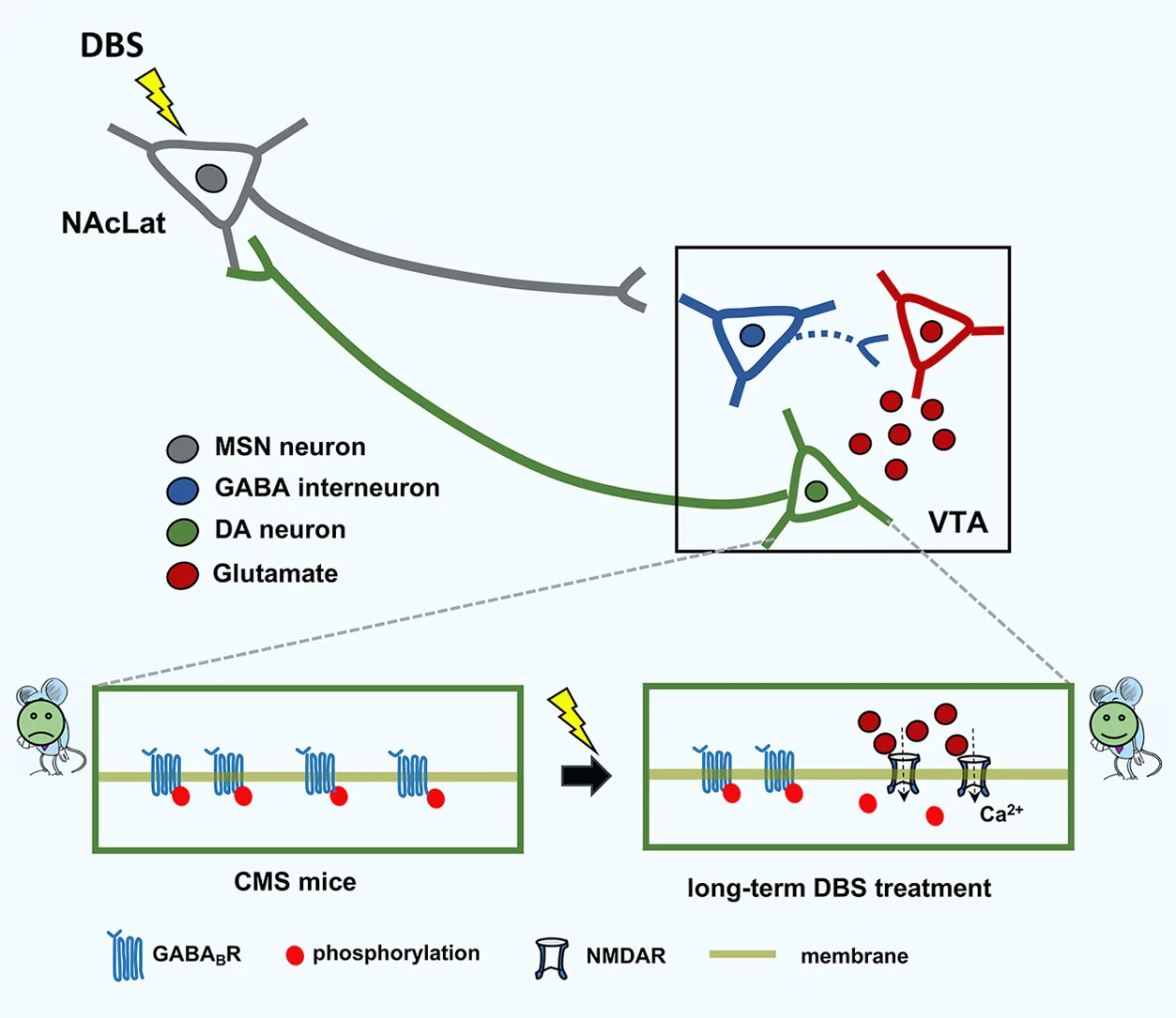

## Introduction

Major depressive disorder (MDD) is the leading cause of disability worldwide [1,2]. Despite available psychosocial and pharmacological interventions, many patients exhibit partial or no response to treatments, resulting in high rates of treatment resistance [3,4]. At the neurophysiological level, decreased spontaneous firing and reduced responsiveness to rewarding stimuli in dopamine (DA) neurons of the ventral tegmental area (VTA) have been consistently observed in CMS model of depression [5,6]. Dysfunction of mesolimbic dopamine system has been strongly implicated in anhedonia, the loss of pleasure or motivation, which represents a core symptom of MDD in both humans and rodents [[7], [8], [9]].

The γ-aminobutyric acid type B receptor (GABA_B_) [10,11] plays a crucial role in mediating prolonged inhibiting control over DA neurons. This slow, metabotropic inhibition regulates dopaminergic excitability and reward responsiveness [[12], [13], [14]]. Notably, repeated exposure to psychostimulants such as methamphetamine reduces GABA_B_-mediated inhibitory currents in midbrain dopamine neurons [15]. Dysfunction of GABA_B_ signaling, particularly excessive inhibition of DA neuronal activity, may therefore underlie anhedonia and motivational deficits in depression.

Deep brain stimulation (DBS) has emerged as an effective neuro-modulatory approach for treatment-resistant depression (TRD). Long-term or repeat high-frequency DBS targeting pathologically overactive circuits improves mood and motivation [[16], [17], [18], [19], [20], [21]]. DBS of the nucleus accumbens (NAc), more than a month, has been approved for obsessive compulsive disorder (OCD) [22] and has shown efficacy in alleviating anhedonia, anxiety and refractory depression [23]. Within the NAc, subregion-specific modulation plays distinct roles [[24], [25], [26]]: activation of the lateral nucleus accumbens shell (NAcLat) enhances positive reinforcement and reward-related behaviors in preclinical models [[27], [28], [29], [30]]. Thus, the NAcLat represents a precise and functionally relevant target for DBS interventions. Previous work has shown that NAc-DBS disinhibits VTA DA neurons by suppressing local VTA GABAergic activity, thereby restoring dopaminergic signaling in CUMS depression model [31]. It is certain that DBS plays a significant role in regulating dopamine function within the reward circuit. However, the mechanisms through which continuous NAcLat-DBS modulates the VTA-NAcLat circuit at the molecular levels remain unclear. Recent evidence suggests that GABA_B_ receptors (GABA_B_) are responsive to neuro-modulatory stimulation such as transcranial magnetic stimulation (TMS) [32]. We therefore hypothesize that NAcLat-targeted DBS exerts its antidepressant effects through GABA_B_-dependent regulation of VTA DA neurons.

To test this hypothesis, this study focuses on the role of DA GABA_B_ in the initiation of NAcLat-DBS and the regulation of DA activity. We employed a chronic mild stress (CMS) mouse model to mimic depressive-like phenotype. Using fiber photometry recording, pharmacological interventions and genetic manipulations in freely moving mice, we examined how NAcLat-DBS alleviates depressive-like behaviors and regulates VTA dopaminergic activity. Furthermore, we investigated whether DA GABA_B_ mediates the antidepressant effects of NAcLat-DBS. These findings provide mechanistic insight into how targeted NAc stimulation restores dopamine-dependent reward processing and mood regulation in depression.

## Material and methods

### Chronic mild stress modeling procedure

Male C57BL/6 J mice (6–8 weeks) were group-housed on a 12 h/12 h light-dark cycle (7 a.m. light-on and 7 p.m. light-off) with ad libitum access to standard chow diet and water in the room that was controlled for temperature and humidity. Mice used in this study were obtained from Beijing Sibeifu Biotechnology Co. LTD with the license number SCXK (Beijing) 2019–0010. These mice were randomly divided into two groups: Control and CMS. The Control mice were reared together, while the CMS mouse was socially isolated by placing each mouse in a single cage and undergoing CMS procedure for five weeks. The chronic stress process was randomly arranged with common mild stressors. The mice received 2 to 3 kinds of stressors every day for five weeks, and the types of stress stimuli every week were as follows: food and water deprivation for 24 h, tilted cage 45° for 8 h, wet cage for 8 h, empty cage for 12 h, constrained for 20 min, strobe light for 8 h, light on for 24 h, forced swimming at 4 °C for 5 min, and tail suspension for 20 min. The stress paradigm was that the same stressor was not applied for 3 consecutive days, the continuous stress for 5 weeks was manipulated, and the Control mice were placed in a separate feeding cage without any contact with the CMS mouse model. All animal experiments complied with the NIH Guide for the Care and Use of Laboratory Animals were approved by the Institutional Animal Care and Use Committee (IACUC) of the Military Medical Research Institute (approval ID: IACUC-JSRZ-23-0201).

Behavioral tests were conducted between 14:00 p.m.–22:00 p.m. Mice were habituated in the behavior testing room for at least 1 h before all the experimental tests. Mice were randomly assigned to be control and experimental groups. Behavioral apparatuses were cleaned and wiped with 75% ethanol to remove odor cues left by the previous subject between trials. Only one behavioral test was conducted each time for all the pharmacological and optogenetic manipulations, and the interval between different tests was at least 3 days.

### Electrodes implantation and deep brain stimulation

Before surgery, 6-week-old mice were handled for 7 days to habituate them to the stimulation procedure and experimenter. Subsequently, we anesthetized the mice intraperitoneally (pentobarbital sodium, 0.7%, 10 μl/μg), and using a stereotaxic frame, implanted electrodes (bipolar, two parallel tungsten wires, twisted, 0.22 mm in diameter) reaching the bilateral NAcLat (coordinates: +1.10 mm anterior, ±1.75 mm lateral, and −4.45 mm ventral to bregma). After 5–7 days of recovery and a 5 weeks' CMS regime, we bilaterally subjected the mice to high-frequency stimulations (130 Hz, 100 μA, 100 μs pulse width), and continuous stimulation was applied simultaneously to both side, 1 h per side each day, with intervention lasting for two consecutive weeks (i.e., NAcLat-DBS). Thus, stimulations took place from the fifth week of CMS, and the CMS mice continued for another 2 weeks’ DBS (i.e., the end of CMS). We controlled current intensity using a constant current isolated stimulator, and synchronized our stimulation protocols using Master-8 (A.M.P·I., Israel). We also placed CMS mice into stimulation chambers daily for 1 h per day without applying any current. Based on stereotaxic targeting parameters and post-hoc histological verification, the electrode tips were tightly confined within the anatomical boundary of NAcLat, not involved in the entire NAc region. All animals with misplaced electrodes, tracks crossing the NAc core, medial NAc, or adjacent striatal areas were excluded from further data analysis.

### Behavioral tests

**Open field test (OFT)** Mice spontaneous activity monitoring system (43.2 cm × 43.2 cm × 30.5 cm) was used to monitor the activity characteristics of mice in natural state. Each mouse was placed in the center of the open area before test and allowed to explore freely for 5 min. After the mice became familiar with the environment, the time spent in the center and total distance traveled within 10 min were recorded and analyzed. The central area is defined as a square of 25% of the total area of OFT. The CMS mouse model presents a reduced total distance and has less time in the central area than the control mice.

**Sucrose preference test (SPT)** Decreased sucrose consumption caused by depression is thought to reflect anhedonia symptom [33]. Before the experiment began, the animals were trained to feed two bottles of water for 36 h, then one bottle of 1% (w/v) sugar solution and one bottle of ordinary water, switching the bottles every 12 h. After the training, the mice were restricted from drinking water for 12 h, then fed a bottle of 1% sugar water and a bottle of regular water for 24 h, still switching the bottles every 12 h. Finally, the sucrose preference is calculated by combining the total consumption of sugar/the total consumption of water and sucrose. There is a decreased sucrose intake (less than 60% in sucrose consumption) in the CMS mice which appeared anhedonia behavior, compared with the control mice.

**Force swimming test (FST)** Mice were placed in a 2 L glass (height 19 cm, diameter 14 cm) of transparent water at a temperature of 21–24 °C and a water depth of 10 cm. The mice were allowed to swim for 6 min, of which 2 min were latency and 4 min were recorded. The behavior of the mice was recorded via a camera. The analysis was then performed using Smart software, which recorded when the mice were floating or stationary as the immobility time; When the immobility time in CMS mice were for twice as long or more than the control mice, the CMS mice are considered to be desperate. There is more immobility time in the CMS mice compared with the control mice, which appeared a despair behavior.

### Tissue processing, immunohistochemistry and imaging

Mice were anesthetized with 0.7% pentobarbital sodium and perfused with 4% paraformaldehyde (PFA) in 0.1 M phosphate-buffered saline (PBS). The brain was dissected from the skull and post-fixed in 4% PFA at 4 °C for 24–48 h. After washing with PBS, the brain was coronally sectioned at 30–40 μm on a freezing microtome (CM1950, Leica). Every three brain slices were chosen and pretreated in 0.2% Triton-X100 in PBS for 1 h at room temperature (RT), then blocked with 0.05% Triton-X100, 10% bovine serum albumin (BSA) in PBS for ∼1.5 h at RT and rinsed in PBS. Brain slices were transferred into primary antibody solution (mouse anti-TH, 1:1000, sc-25269, Santa Cruz; rabbit anti-GABA_B1_R, 1: 600, ab238130, Abcam; rabbit anti-c-Fos, 1:1000, Synaptic System) in PBS with 0.3% Triton-X100 and incubated overnight at 4 °C. Tissues were rinsed in PBS three times, and incubated with secondary antibody solution (goat anti-rabbit 488, 589, goat anti-mouse 589, 1:2000, Life Technologies) in PBS for 1.5 h at RT, then rinsed and mounted onto slides, dried and covered under glycerol: TBS (3:1) with Hoechst 33,342 (1:1000, Thermo Fisher Scientific). Sections were imaged with a Nikon-A1 (Tokyo, Japan) slide scanning confocal microscope with 20 × objectives.

### In situ hybridization followed by immunohistochemistry

Mice were deeply anesthetized with 0.7% pentobarbital (10 μl/μg, i. p.) and perfused with 0.9% saline. Brains were quickly removed at 4 °C and stored at −80 °C. The brains were returned at −20 °C for 60 min, and the fresh brains were cut serially (15 μm) on a cryostat at −20 °C (CM1950, Leica) and mounted directly onto super frost glass slides (Fisher). Sections were stored at −80 °C before starting the RNAscope assay (Advanced cell diagnostics, ACD). Briefly, before hybridization, the free-floating sections were incubated in 10% formalin for 60 min, and gradient dehydration in 50%, 70%, 100%, 100% ethanol each for 5 min. Slides were washed twice in PBS for 2min, and once in 100% ethanol before incubation with protease IV (ACD) at 40 °C for 30 min. RNA hybridization was performed according to the fluorescent multiplex kit (ACD) using an antisense probe against (ACD 319171-C1). After the last wash step, slides were rinsed two times in PBS, blocked in 5% normal donkey serum/0.2% Triton X-100 in PBS for 1 h at room temperature and incubated with primary antibodies (^TM^Probe-TH 1:300; AB152, Chemicon; mouse-anti-Gabbr1 1:50; MAB369, Chemicon; ^TM^Probe-Slc32a1-C2 1:300) overnight at 40 °C in blocking buffer. Next day, slides were washed three times in PBS for 15 min and incubated with secondary antibodies (1:10; Alexa Fluor 520 and 650; Jackson Immuno-research) for 2 h at room temperature. Finally, slides were washed three times in PBS for 15 min and cover slipped using DAPI-containing fluor mount-G. Images were taken at 60 × magnification using a Nikon-A1 (Tokyo, Japan) confocal microscope. For cell counting quantification, sections covering from the rostral to caudal extent of the VTA were collected and stained for TH/Slc32a1-C2 (GABA) and Gabbr1. For RNAscope quantification of gabbr1-positive DA/GABA neurons, the gabbr1 immunofluorescence intensity was measured by Image J. Taking one out of every three slices [3,4] in VTA sections was selected to calculate gabbr1 immunofluorescence intensity per mouse by an experimenter double-blind to the treatment.

### Tissue collection and membrane protein extraction

Mice were deeply anesthetized and perfused with ice-cold 0.9% saline. Their brains were quickly removed and placed on ice. The VTA was dissected from the brain using a mouse brain matrix (RWD life science), and tissue punches (Harris Uni-Core) were obtained according to stereotaxic brain atlas [34]. The freshly isolated tissue was processed for membrane protein extraction. The membrane protein extracts were isolated using the Thermo Scientific Mem-PER Plus Membrane Protein Extraction Kit (No. 89842Y) following the manufacturer's instructions. Brain tissue was initially washed in the cell wash solution and homogenized in the permeabilization buffer using a Dounce tissue grinder. The homogenate was transferred to a new 2 ml Eppendorf tube, incubated at 4 °C for 10 min under constant mixing, and cytosolic proteins were separated via centrifugation at 16,000×*g* for 15 min at 4 °C. The pellet was resuspended in the solubilization buffer and incubated at 4 °C for 30 min under constant mixing. The membrane and the membrane-associated proteins were obtained via centrifugation of 16,000×*g* for 15 min at 4 °C.

### Western blot analysis

The protein concentration was determined using a BCA protein assay kit (Thermo Scientific). Protein extracts were separated by electrophoresis on an 8% SDS-PAGE gel (CWBIO, China) and then transferred onto polyvinylidene fluoride membranes. The membranes were incubated consecutively with primary and secondary antibodies, enhanced chemiluminescence solution, and subjected to auto-radiography. The goat anti-GIRK2 (1:1000), mouse anti-GABA_B1_R (1:1000), and rabbit anti GABA_B2_R (1:1000) and anti-pan-cadherin (1:1000) antibodies were purchased from Abcam (Abcam, Cambridge, MA, USA). The horseradish peroxidase secondary antibodies (1:1000) were obtained from Cell Signaling Technology (Boston, MD, USA). For phosphorylation detection, the rabbit anti-*p*-GABA_B2_R-s783 (1:10) antibody was purchased from Abcam (ab72447). The intensities of the blots were analyzed with Image Pro Plus 6.0.

### In vivo stereotaxic intracranial and optical fiber implantation

Mice (6 weeks) were intraperitoneally anesthetized with 0.7% pentobarbital (10 ml/kg) and placed in a stereotaxic apparatus (68,018, RWD). For virus injections, recombinant AAVs were delivered into VTA and NAcLat by a micro-syringe pump controller (LEGATO 130 kd Scientific Company) at a volume of 200–400 nl with a flow rate of 40 nl/s. Injection pipette was left at the injection site for 5–10 min after viral delivery to assure adequate viral delivery. The following coordinates were used: VTA (3.15 mm posterior to bregma, 0.5 mm lateral to the midline, 4.35 and 4.45 mm below the pia), NAcLat (1.10 mm anterior to bregma, 1.75 mm lateral to the midline, 4.45 mm below the pia). Coordinates were slightly adjusted based on mouse age and brain size. For in vivo optogenetic stimulation, an optical fiber (200 μm diameter, 0.37 numerical aperture; RWD) was implanted above the VTA and NAcLat using stereotaxic guidance following a minimum 3-week post-injection recovery period. Under isoflurane anesthesia, the chronically implanted optical fiber (Shanghai Fib-laser) was secured to the skull using a dental cement cap that incorporated the fiber optic cannula and its protective sleeve. Following this, a post-procedural recovery period of at least 30 min was provided in the home cage. An optogenetic system stimulator (IOS-589 RWD) (blue light: 473 nm; yellow light: 589 nm; 5 mW; 15 ms pulse duration; 20 Hz) was delivered to modulate VTA-derived inputs to the NAcLat. The same optical delivery parameters were applied to control mice. The following AAV viruses were used from the BrainVTA and Braincase Biotechnology in this experimental method: AAV_2/5_-mTH-NLS-Cre-WPRE-SV40 polyA (3.4 × 10^12^ vector genomes/l), AAV_2/9_-hSyn-DIO-ChrimsonR-mCherry-WPRE-hGH pA (1.48 × 10^12^ vector genomes/ml), AAV_2/5_-hSyn-DA4.4 (1.66 × 10^13^ vector genomes/ml), AAV_9_-hSyn-iGluSnFR3. v857. GPI (2.0 × 10^12^ vector genomes/ml), AAV_9_-mDlx-GCAMP6s (2.0 × 10^12^ vector genomes/ml), AAV_9_-CAMKIIa-GCAMP6s (2.0 × 10^12^ vector genomes/ml) and AAV_2/9_-TH-GCAMP6s (2.0 × 10^12^ vector genomes/ml). AAV_retro_-hSyn-EGFP-3XFlag-WPRE-hGH pA (4.2 × 10^12^ vector genomes/ml).

### Transfection of Gabbr1 gene and Gabbr1-RNAi virus in the DA neurons

The gabbr1 gene sequence was screened as NM_019,439 by PubMed NBCI primer design. Construction of the AAV vector of gabbr1 (GABA_B1_) gene adeno-associated virus was commissioned through BrainVTA Biotechnology company (Wuhan, China), and the virus was constructed as AAV_2/9_-TH-Gabbr1-P2A-mCherry-WPREs/AAV_2/9_-TH-mCherry-WPREs. The construction of the gabbr1 (GABA_B1_) gene RNAi interfering adeno-associated virus AAV vector was commissioned through Jikai Biotechnology Co. LTD. The interference virus was designed by using the TH-activated external screen effective target, and the virus element sequence was TH promotor-EGFP-mir155 (MCS)-SV40 polyA. The virus is constructed as AAV_9_-TH-Gabbr1-RNAi-EGFP/AAV_9_-TH-Gabbr1-NA-EGFP. The animals were divided into four groups before surgery: TH-mCherry, TH-Gabbr1-mCherry, TH-Gabbr1-NA and TH-Gabbr1-RNAi groups. After four weeks of virus expression and CMS processing, the depressive-like behavior in all groups were monitored, and then brain perfusion was used to detect gabbr1 expression or interference in VTA area. The following AAV viruses were used from the BrainVTA and Braincase Biotechnology in this experimental method: AAV_9_-TH-Gabbr1-NA-EGFP (1 × 10^12^ vector genomes/ml, Jikai), AAV_9_-TH-Gabbr1-RNAi-EGFP (1 × 10^12^ vector genomes/ml, Jikai), AAV_2/9_-TH-mCherry-WPREs (5 × 10^12^ vector genomes/ml), AAV_2/9_-TH-Gabbr1-P2A-mCherry-WPREs (5 × 10^12^ vector genomes/ml).

### Cannula implantation

For in vivo photo-stimulation experiments, an optical fiber (200 μm, 0.37 NA, RWD) was implanted into the VTA and the NAcLat, using stereotaxic apparatus at least 3 weeks after viral injection surgery. For drug microinjection, mice were implanted with a stainless-steel double catheterization cannula (core spacing width:1 mm) bilaterally above the VTA. Stainless dummies were inserted into the guide cannula to prevent the guide cannula from clogging. Dental cement was used to secure optical fibers and cannula to the skull. For electrode implantation, mice were implanted with a homemade copper wire electrode bilaterally above the NAcLat. At least 1 week after fiber or cannula implantation surgery, vehicle (aCSF) or drug (CGP35348 4 mM, 1 μl/Baclofen 5 nM, 1 μl) was delivered into the VTA via an injection cannula that extended 1.0 mm beyond the tip of the guide cannula [35]. At least 1 week after electrode implantation surgery, CMS modeling procedure began. A volume of 1 μl vehicle or drug was injected over a 5 min period. The injection cannula was kept in the injection place for an additional 5 min to minimize the drug leaking. The baclofen (3 mg/kg), a GABA_B_ agonist was intraperitoneally injected for 7 days to observe the effect of baclofen on depressive-like behaviors in normal mice. CGP35348 (1 mg/kg), a GABA_B1_ antagonist was injected intraperitoneally, and the control was injected intraperitoneally with the same amount of saline for 14 days (at 14:00 p.m.) to observe the effects of CGP35348 on depressive-like behaviors in the Control and CMS mice. Behavioral tests were conducted around 30 min after microinjection. Brains were sectioned to verify the cannula/electrode location and injection site after finishing all the behavioral tests. Mice with wrong implantation locations were excluded from analyses.

### Data analysis

In this study, no statistical method was used to pre-determine sample sizes, and the sample sizes were determined based on previous studies conducted in the same field. All behavioral data analyses were performed using Graphpad prism (8.0). For homogeneous variances, two-tailed *t-test* and paired/unpaired *t-test* were used for single and paired comparisons, a two-way repeated-measures ANOVA followed by post hoc Tukey's test or two-way ANOVA followed by Sidak's multiple comparison test was used for multiple comparisons. *P < 0.05* was considered statistically significant.

## Results

### NAcLat-DBS markedly reduced DA GABA_B1_ expression and reversed dopamine neuronal activity in CMS mouse model

First, chronic mild stress (CMS)-induced depression model was established (Supplementary Fig. 1, Fig. 1A). The depression symptoms were assessed by well-documented anhedonia for sucrose preference test (SPT), and despair for force swimming test (FST), as well as locomotor activity for open field test (OFT). Relative to the Control mice, CMS mice exhibited reduced sucrose preference and a significant increase in immobility time in FST. During two-week's DBS treatment (3 days, 7 days, 10 days and 14 days), the sucrose consumption rose in CMS mice, while their immobility time gradually decreased as the treatment progressed (Fig. 1B and C). Notably, the maximum reduction in immobility time was observed on the 10th day of DBS intervention, which markedly alleviated despair-like behaviors in CMS mice (Fig. 1D). Dopamine (DA) release was measured in the CMS, CMS-DBS and Sham mice by recording fluorescence dynamics of DA4.4, a DA sensor (Fig. 1L–M).We observed a concordant relatively low DA sensor fluorescence in the NAcLat when activating VTA-DA neurons (laser-on) in the CMS mice, while activating Sham mice induced DA release at a higher level than the CMS mice (10.02 *vs* 2.03, % Δ F/F, *p < 0.001*) in the same baseline level (Fig. 1N–O). These data show that decreased DA release in NAcLat from the terminals of activated DA is crucial for depression pathological state. Second, the CMS mice were implanted bilaterally with electrodes for DBS treatment at 130 Hz (1 h/day at 100 μA, 100 μs) for two weeks (Supplementary Fig. 2A–B), with the contact point in the NAcLat (Fig. 1I). It was found that there is a remarkably increased DA release (3.42 *vs* 1.74, % Δ F/F, *p < 0.01*) in the same baseline level and an increased c-Fos expression (Fig. 1P–Q, Supplementary Fig. 2C) in the CMS-DBS mice in comparison with CMS mice. In addition, to investigate that whether GABA_B_ participated in the molecular mechanism of DBS, the GABA_B1_ expression was assessed in the CMS-DBS, CMS and Control mice. In situ hybridization (ISH) was performed to evaluate GABA_B1_ (gabbr1) mRNA expression. We found that after DBS treatment, the gabbr1 mRNA expression remained unchanged in GABA neurons in the CMS mice (Fig. 1F and H). Surprisingly, immunofluorescence (IF) and ISH results showed that decreased GABA_B1_ and gabbr1 mRNA expression in DA neurons were observed in the CMS-DBS mice compared with the CMS mice (Fig. 1E and G, Supplementary Fig. 2D and E). These results implied that DBS caused reduced GABA_B1_ expression in VTA-DA neurons in CMS model mice. Further, immunoblot has verified in the Control, CMS and CMS-DBS mice that there is no significant difference among them on the total expression of GABA_B1_ during two-week's DBS treatment (Fig. 1J–K). Overall, these results suggested that long-term DBS NAcLat greatly has relieved depression behaviors induced by chronic mild stress, in which the antidepressant effects of DBS is potentially associated with the down-regulated GABA_B1_ expression in VTA DA neurons.

**Fig. 1:**
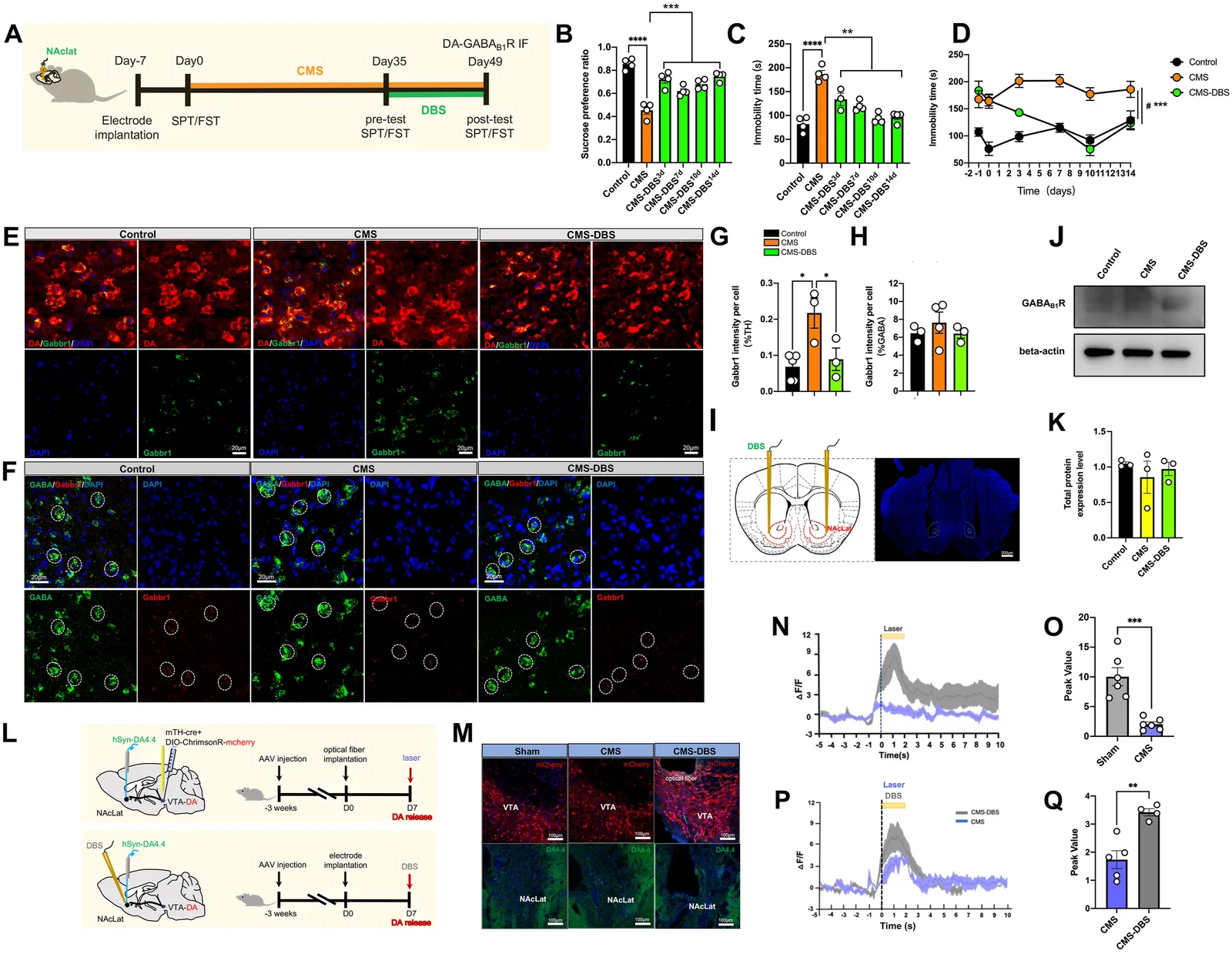
**NAcLat-DBS reduced GABA_B1_ expression of dopamine neurons and revived dopamine neuronal activity in the CMS mouse model** (A) Experimental process of long-term DBS treatment in CMS mouse model. Mice were bilaterally implanted with DBS electrode in the NAcLat, and the baseline behaviors were tested 7 days later. CMS mouse model was established for five weeks. Long-term DBS (at 130 Hz, 100 μA, 100 μs pulse width) treatment for two weeks. (B)–(D) DBS (0 day, 3 days, 7 days, 10 days, 14 days) alleviated CMS mice's depressive-like behaviors in sucrose preference (B) and force swimming tests (C, D). (E) and (F) Brain slices immunostaining was carried out after long-term DBS treatment in RNAscope for Gabbr1 mRNA expression in dopamine and GABA neurons in Control, CMS and CMS-DBS groups (scale bars = 20 μm). (G) The Gabbr1 mRNA intensity in the DA neurons of Control, CMS and CMS-DBS groups. (H) The Gabbr1 mRNA intensity in the GABA neurons of Control, CMS and CMS-DBS groups. (I) Electrode implantation in bilateral NAcLat, scale bars = 200 μm. (J) The relative expression of total GABA_B1_R protein in the VTA area; (K) The total GABA_B1_ protein expression in immunoblot assay about Control, CMS and CMS-DBS mice. (L) Schematic of virus injection, fiber photometry and DBS recordings of the dopamine neurotransmitter release task. (M) Representative coronal images showed the mCherry expression in the DA neurons of VTA and DA4.4 expression in the NAcLat. Red: mCherry, Green: DA4.4, Blue: DAPI. Scale bars = 100 μm. (N) Average ΔF/F of DA4.4 fluorescence in response to optical stimulation in the Sham and CMS mice. (O) The peak value comparison in the Sham and CMS groups. (P) Average ΔF/F of DA4.4 fluorescence in response to optical stimulation and DBS in the CMS and CMS-DBS mice. (Q) The peak value comparison in the CMS and CMS-DBS groups. All data are expressed as mean ± SEM. Statistical analysis was performed using two-way repeated-measures ANOVA followed by post-hoc tests analysis for (B)–(D), n = 4. *∗∗∗∗p* < 0.0001, CMS *vs*. Control, *∗∗∗p* < 0.001, *∗∗p* < 0.001, CMS-DBS^3d−14d^*vs.* CMS; One-way ANOVA analysis for (G) and (H), n = 3. *∗p* < 0.05, CMS *vs*. Control, CMS-DBS *vs*. CMS; One-way ANOVA analysis for (K), n = 7, and Unpaired *t-test* analysis for (O) and (Q), n = 6 and n = 4. ∗∗∗*p* < 0.001, CMS *vs.* Sham; *∗∗p* < 0.01, CMS-DBS *vs.* CMS.

**Fig. 2:**
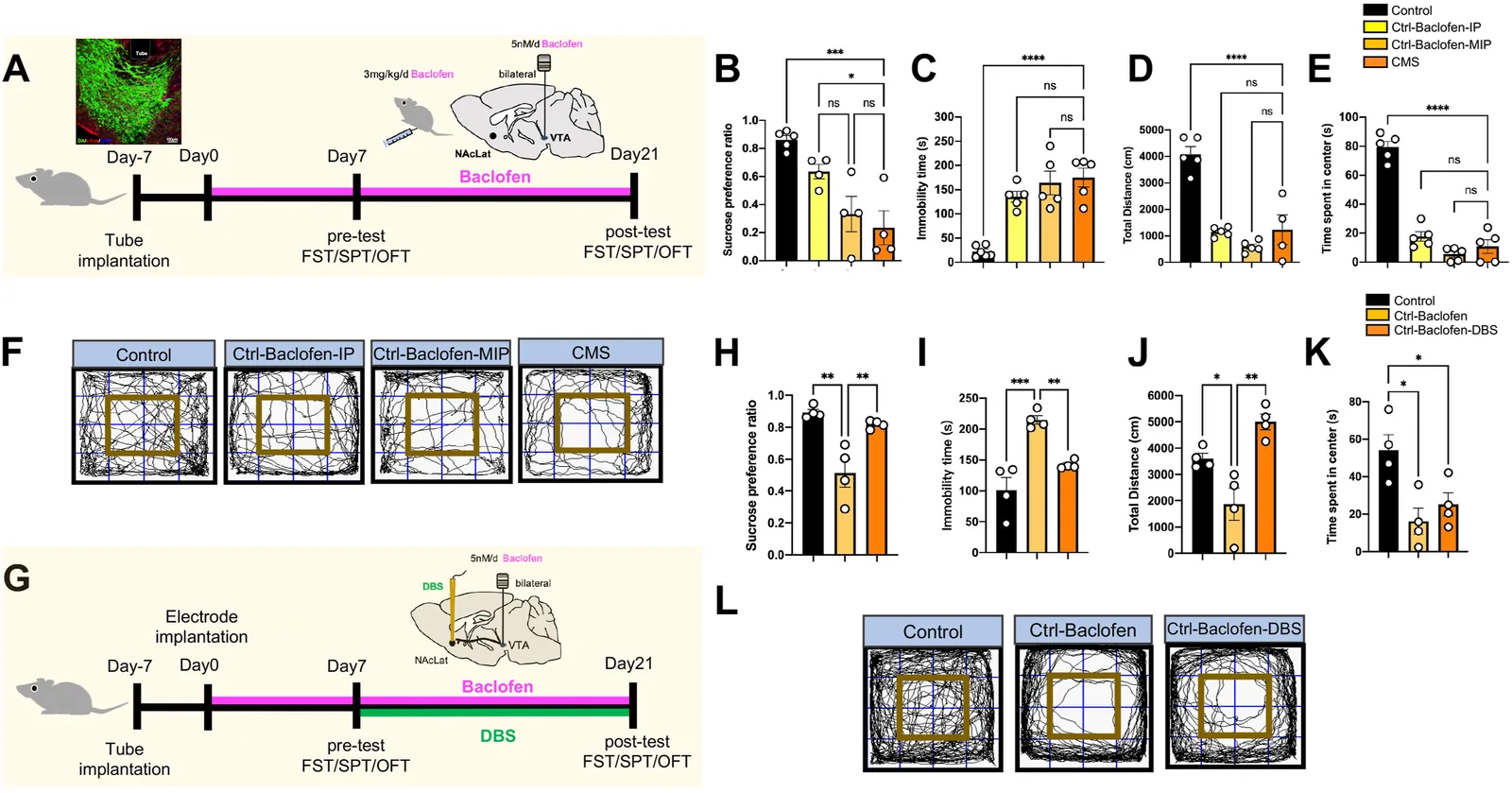
**NAcLat-DBS rescued the depressive-like behaviors induced by baclofen** (A) Experimental scheme of injection intraperitoneally with Baclofen (3 mg/kg/d) or the VTA infusion Baclofen (5 nMol/d) via tube for 21 Days and representative coronal images showed the tube location in the VTA. Green: TH (DA), Red: c-Fos, Blue: DAPI, scale bar = 100 μm. (B)–(E) The 24 h sucrose preference ratio, immobility time, travel distance and center exploration time were tested in the Control, Ctrl-Baclofen-IP (intraperitoneal injection, IP), Ctrl-Baclofen-MIP (micro-injection pump, MIP) and CMS groups. (F) Trajectory diagram in the Control, Ctrl-Baclofen-IP, Ctrl-Baclofen-MIP and CMS groups. (G) Schematic of tube and electrode implantation and NAcLat-DBS bilaterally was carried out during two weeks' baclofen administration. (H–K) The 24 h sucrose preference ratio, immobility time, travel distance and center exploration time were tested in the Control, Ctrl-Baclofen (Ctrl-Baclofen-MIP) and Ctrl-Baclofen-DBS groups. (L) Track diagram of spontaneous activity in the Control, Ctrl-Baclofen and Ctrl-Baclofen-DBS groups. All data are expressed as mean ± SEM. One-way ANOVA analysis for (B)–(E) and (H)–(K), n = 4. *∗∗∗p* < 0.001, *∗∗∗∗p* < 0.0001, CMS *vs*. Control; *∗p* < 0.05, CMS *vs*. Ctrl-Baclofen-IP; and (H)–(K), *∗∗p* < 0.01, *∗∗∗p* < 0.001, *∗p* < 0.05, Ctrl-Baclofen *vs*. Control, ∗∗*p* < 0.01, Ctrl-Baclofen-DBS *vs*. Ctrl-Baclofen, *∗p* < 0.05, Ctrl-Baclofen-DBS *vs*. Control.

### NAcLat-DBS rescued the despair and anhedonia behaviors resulting from pharmacological activation of GABA_B_ in normal mouse

To determine whether there is a direct correlation between long-term DBS and DA GABA_B_, by intraperitoneal injection (IP) and micro-perfusion (MIP) baclofen into the bilateral VTA respectively in the normal mice (Fig. 2A), GABA_B_ was selectively activated. After tube implantation for one week, the mice were carried out baclofen administration. Baclofen treatment three weeks later, it was found that the normal mice were induced depressive-like symptoms. Compared with the Control group, mice in the Ctrl-Baclofen-IP and the Ctrl-Baclofen-MIP groups have showed decreased sucrose consumption in the SPT (Fig. 2B) and increased immobility time in the FST (Fig. 2C), which manifested as anhedonia and despair behaviors during three weeks' baclofen administration. Furthermore, the baclofen induced decreased central exploration time and total distance in the Ctrl-Baclofen-IP and the Ctrl-Baclofen-MIP groups relative to the Control group in the OFT (Fig. 2D–F). In above behaviors assay, there have few differences among Ctrl-Baclofen-IP, Ctrl-Baclofen-MIP and CMS groups (positive control). Thus, lasting activation of GABA_B_ in the VTA enhanced susceptibility to depression, leading to depressive-like behaviors in the normal mice. In addition, we have further evaluated that whether long-term DBS NAcLat alleviates the depressive-like behaviors of baclofen-mice in the VTA-NAcLat pathway (Fig. 2G). While continuing the administration of baclofen, two-week's DBS treatment was carried out. We found that lasting DBS rescued the reduction of 24 h sucrose consumption and the increased immobility time in the baclofen mice (Ctrl-Baclofen-IP and Ctrl-Baclofen-MIP) (Fig. 2H and I). Similar to the CMS mice, DBS corrected the declines of total distance traveled and the central exploration time in the OFT in baclofen mice (Fig. 2J-L). These results suggest that long-term DBS rescued baclofen-induced depression symptoms, hinting GABA_B_ involves in the mechanism of long-term DBS effects in mesolimbic VTA-NAcLat pathway.

### Pharmacological inhibition GABA_B_ receptor played a major role on alleviating despair and anhedonia symptoms in CMS mouse model

To further evaluate the effects of inhibiting GABA_B_ on depression phenotypes, CGP35348, a GABA_B_ antagonist, was intraperitoneal injected (1 mg/kg/d) or bilaterally infused (4 mM/d) into the VTA for two weeks in the CMS mice. The CGP35348 was intraperitoneally injected in 15:00 p.m. every day in the Control and CMS mice respectively (Fig. 3A, Supplementary Fig. 4A). During CGP35348 administration, the behaviors were observed in the third day, seventh day, tenth day and fourteenth day. Following CGP injection, the percentage of 24 h sucrose consumption has gradually increased and the sucrose consumption reached the maximum during 7–10 days, which exhibited clearly difference compared with the Control mice (Fig. 3B). Moreover, the immobility time in the FST gradually decreased during CGP treatment, and it is close to the control mice during 7–10 days in the CMS mice (Fig. 3C). In addition, the time spent in center gradually increased (Supplementary Fig. 4D) and the total distance remained unchanged (Supplementary Fig. 4B–C) in the CMS mice, however, there were still differences between the CMS and Control mice following CGP administration (Supplementary Fig. 4). After VTA CGP/Saline perfusion for 14 days (Fig. 3D), the SPT and FST results showed that there have no effects on the Control-CGP and Control-saline mice, but the 24 h sucrose consumption increased and the immobility time reduced after CGP35348 treatment in the CMS mice compared with the CMS-saline mice (Fig. 3E–F). Furthermore, the total distance traveled and center exploration time in the OFT have an increase in the CMS-CGP mice relative to the CMS-saline mice, but there was no obvious difference between the Control-CGP and Control-saline mice (Fig. 3G–I). These results indicated that the CMS mice's depression symptoms were corrected by continuous GABA_B_ inhibition. Whatever received saline or CGP, there were no clear differences in the Control mice, and there are always difference between the CMS mice and the Control mice in behaviors detection. These findings suggest application of GABA_B_ antagonist alleviated CMS mice's depressive-like symptoms consistent with the long-term DBS.

**Fig. 3:**
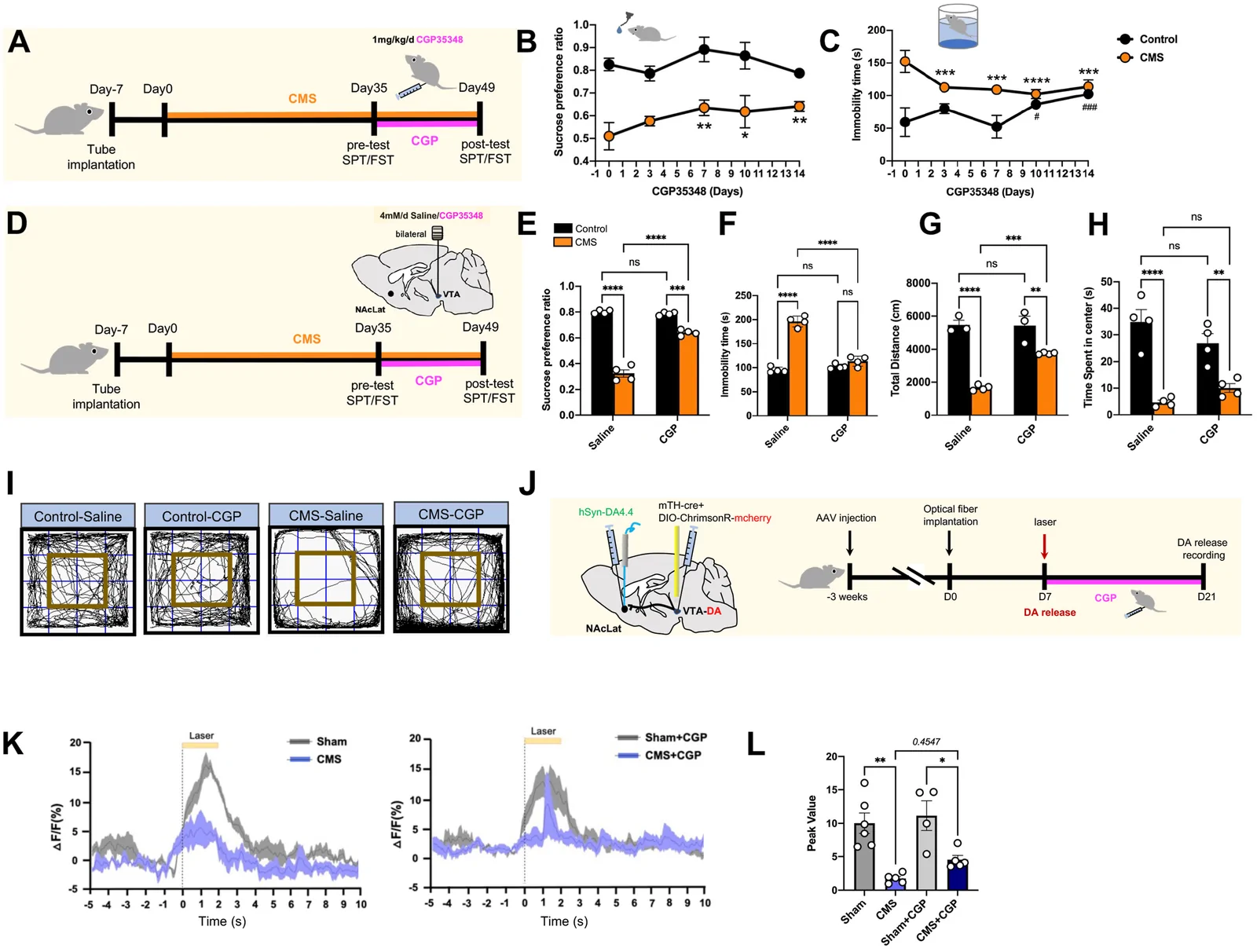
**GABA_B_ receptor inhibition alleviated depressive-like symptoms in CMS mouse model** (A) Experimental process of intraperitoneal injection CGP35348 (1 mg/kg/d) for fourteen days in Control and CMS mice and the SPT and FST were carried out. (B) Line graph: the change of 24 h sucrose preference ratio during CGP administration in Control and CMS groups. (C) Line graph: the change of force swim immobility time during CGP administration in Control and CMS groups. (D) Experimental scheme of the VTA infusion Saline/CGP35348 (4 mMol/d) bilaterally via tube for fourteen days and the SPT and FST were carried out. (E) The 24 h sucrose preference ratio in Control-Saline, Control-CGP, CMS-Saline and CMS-CGP groups. (F) Immobility time in the Control-Saline, Control-CGP, CMS-Saline and CMS-CGP groups. (G)–(H) total distance and time spent in center in the Control-Saline, Control-CGP, CMS-Saline and CMS-CGP groups. (I) Track diagram of spontaneous activity in the four groups. (J) Schematic of virus injection and fiber photometry recordings of DA4.4 (589 nm, 20 Hz, 5 ms pulse width, 2 s duration, 40 s interval, 10 trials) in Sham, CMS, Sham + CGP and CMS + CGP groups, and neurotransmitter dopamine release task. (K) Left: average ΔF/F of DA4.4 fluorescence in response to optical stimulation of Sham and CMS mice; Right: after intraperitoneal CGP administration, the average ΔF/F of DA4.4 fluorescence in response to optical stimulation of Sham and CMS (Sham + CGP and CMS + CGP) mice. (L) The peak value comparison in the Sham, CMS, Sham + CGP and CMS + CGP groups. All data are expressed as mean ± SEM. Repeated-measures ANOVA followed by post-hoc tests analysis for (B) and (C), n = 4. *∗∗p* < 0.01, CMS-CGP^7d, 14d^*vs*. CMS-CGP^0d^, *∗p* < 0.05, CMS-CGP^10d^*vs*. CMS-CGP^0d^. ^*#*^*p* < 0.05, Control-CGP^10d^*vs*. Control-CGP^0d^, ^*###*^*p* < 0.001, CMS-CGP^14d^*vs*. CMS-CGP^0d^, *∗∗∗p* < 0.001, CMS-CGP^3d,^^7d, 14d^*vs*. CMS-CGP^0d^, ∗∗∗∗*p* < 0.0001, CMS-CGP^10d^*vs*. CMS-CGP^0d^. Two-way ANOVA was used for analysis of panels (E)–(H), n = 4. *∗∗∗∗p* < 0.0001, CMS-Saline *vs.* Control-Saline, *∗∗∗p* < 0.001, *∗∗p* < 0.01, CMS-CGP *vs*. Control-CGP, *∗∗∗∗p* < 0.0001, *∗∗∗p* < 0.001, CMS-CGP *vs*. CMS-Saline, and (L), n = 4. *∗∗p* < 0.01, CMS *vs*. Sham, *∗p* < 0.05, CMS + CGP *vs*. Sham + CGP, *p* = 0.4547, CMS + CGP *vs*. CMS.

In the mesolimbic VTA-NAcLat pathway, whether inhibiting the GABA_B_ expression alleviates the decreased dopamine activity (Fig. 3J). Hence, we first used optogenetic method to directly manipulate the activity of VTA DA neurons with 589 nm laser combing AAV-mTH-cre and AAV-hSyn-DIO-ChrimsonR-mCherry virus expression and further measured the dopamine release in normal mice and CMS model mice (Fig. 3J–K). Optogenetic activating VTA DA neurons with ChrimsonR-mCherry expression appear a significant reduced transmitter release in CMS mouse model compared with the Sham mice (% Δ F/F, 10.02 *vs*. 1.74, *p < 0.01*) (Fig. 3L). Moreover, we also monitored the variation of dopamine transmitters during GABA_B_ inhibition in both groups. Surprisingly, in vivo inhibition of GABA_B_ by intraperitoneal (i.p.) injection of CGP35348 present a partial remission on the DA transmitter in CMS mice despite the absence of statistically significant differences (CMS + CGP *vs*. CMS, % Δ F/F, 4.54 *vs.* 1.74, *p=0.4547*) (Fig. 3L). It was found that after inhibition of GABA_B_ in the Sham group, there was no significant change on the dopamine transmitter release (Sham + CGP *vs*. Sham, % Δ F/F, 11.15 *vs.* 10.02) (Fig. 3L). In addition, we have observed the effects of specifically interfering with GABA_B_ in VTA dopamine neurons on depressive behavior (Supplementary Fig. 3G and H). The results present that TH-Gabbr1-RNAi group was more resilient to depression than the Control and TH-Gabbr1 groups, and the immobility time of force swimming was reduced after interference with VTA-DA GABA_B1_ (Supplementary Fig. 3I), and the central exploration time and the total distance were increased in TH-Gabbr1-RNAi group (Supplementary Fig. 3J-L). Together, inhibiting GABA_B_ alleviated despair and anhedonia symptoms in CMS mice.

### Overexpression DA GABA_B1_ in the VTA-NAcLat pathway antagonized the antidepressant effects of long-term DBS

We next dissected the influence of specific overexpression GABA_B1_ in DA neurons for CMS mice on long-term DBS treatment. Firstly, the AAV_retro_-TH-Gabbr1 virus was bilaterally transfected into the NAcLat respectively in CMS mice, in which overexpression gabbr1 gene retrograde to upstream DA neurons. The Control mice were only transfected AAV_retro_-TH-mCherry into the NAcLat and mCherry gene was expressed in the DA neurons (Fig. 4A). Immunofluorescent staining assay was utilized to verify the relative expression of GABA_B1_/mCherry gene in CMS^TH-mCherry^/CMS^TH−Gabbr1^ mice (Fig. 4E). We found that the CMS^TH−Gabbr1^ mice which suffered from chronic mild stress for three weeks, present remarkable depression phenotypes compared with the CMS^TH-mCherry^ and Ctrl^TH-mCherry^ mice in SPT and OFT, suggesting that high GABA_B1_ expression of DA neurons enhances depression susceptibility in CMS^TH−Gabbr1^ mice more than CMS mice undergoing chronic mild stress for five weeks (Fig. 4B–D, F). Furthermore, following long-term DBS treatment (Fig. 4G), the depressive-like symptoms of CMS^TH−Gabbr1^ mice were not rescued, implying that overexpression DA GABA_B1_ reverse the antidepressant effects of DBS. There have obvious differences between the CMS^TH−Gabbr1^ mice and CMS mice following chronic mild stress for three weeks including 24 h sucrose consumption in the SPT (Fig. 4H), total distance traveled and center exploration time in the OFT (Fig. 4I–K). Further, we have also observed the effects of specifically overexpression DA GABA_B1_ on depressive behaviors (Supplementary Fig. 3A and B). The results showed that the forced swim immobility time increased (Supplementary Fig. 3C), the total distance and center exploration time in the open field decreased in the CMS^TH−Gabbr1^ group compared with the Control and CMS^TH-mCherry^ groups after specific overexpression of GABA_B1_ in dopamine neurons (Supplementary Fig. 3D–F), which appeared a susceptible depressive-like behavior. These experiments indicate that increased gabbr1 expression in the DA neurons contributes to withstand the antidepressant effects of long-term DBS.

**Fig. 4:**
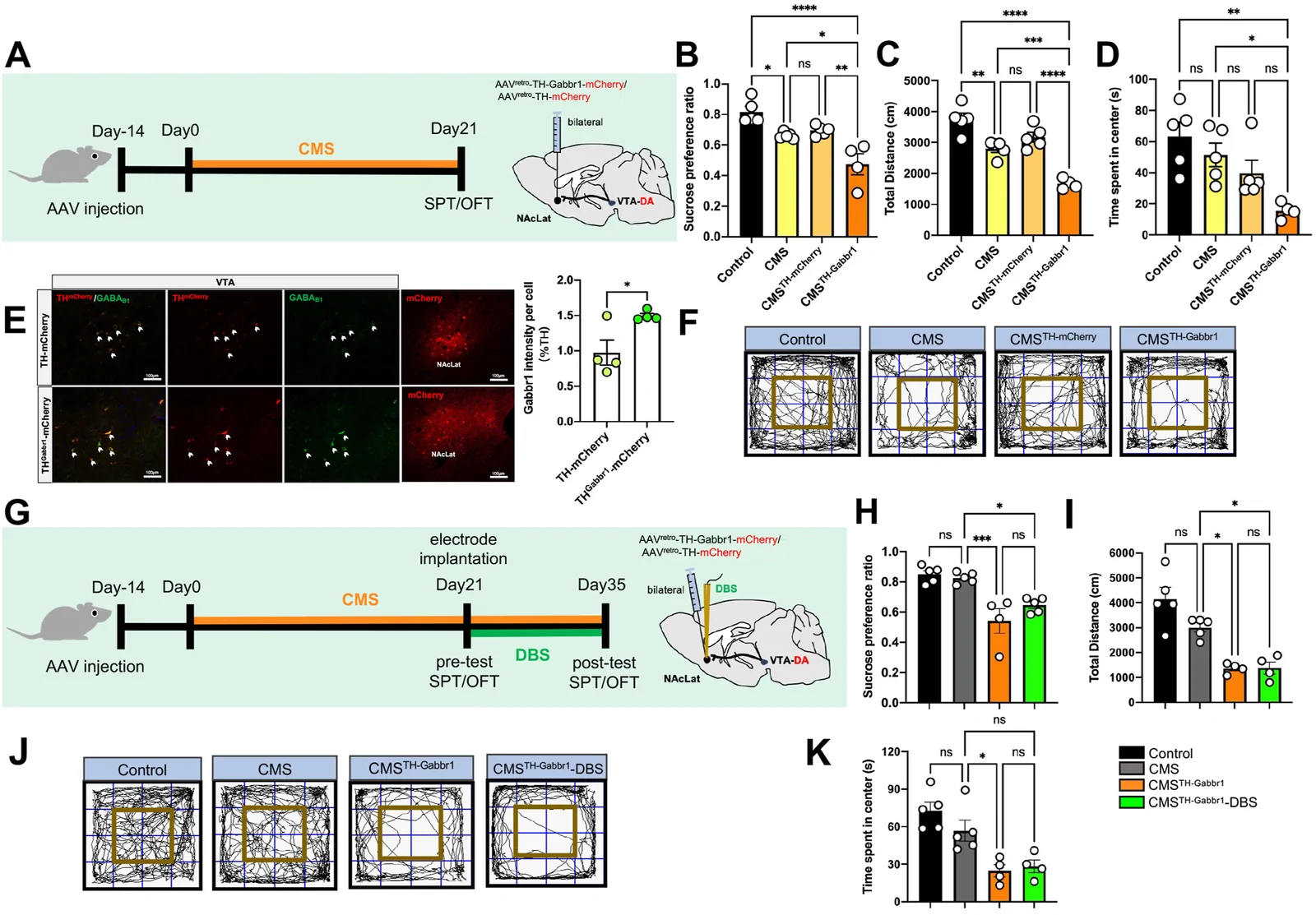
**Overexpression DA GABA_B1_ in the VTA-NAcLat pathway reverse the antidepressant effects of DBS in CMS mouse model** (A) Experimental scheme of overexpression GABA_B1_ in dopamine neurons in the VTA-NAcLat pathway, and schematic of virus injection of AAV_retro_-TH-Gabbr1-mCherry or AAV_retro_-TH-mCherry in bilateral NAcLat. (B)–(D) The 24 h sucrose preference ratio, travel distance and center exploration time were tested in the Control, CMS, CMS^TH-mCherry^ and CMS^TH−Gabbr1^ groups. (E) Left: representative images of the VTA: the GABA_B1_^+^ intensity in dopamine cells in the VTA. Red: TH^mCherry^/mCherry in the NAcLat; Green: GABA_B1_ (Gabbr1), scale bars = 100 μm; Right: Gabbr1 intensity in the DA neurons of TH-mCherry and TH^Gabbr1^-mCherry groups. (F) Trajectory diagram in the Control, CMS, CMS^TH-mCherry^ and CMS^TH−Gabbr1^ mice. (G) Schematic of virus injection on overexpression DA GABA_B1_ in the VTA-NAcLat pathway and electrode implantation in the bilateral NAcLat. (H)–(I) The 24 h sucrose preference ratio, travel distance and center exploration time were tested in the Control, CMS, CMS^TH−Gabbr1^ and CMS^TH−Gabbr1^-DBS groups. (J) Trajectory diagram in the Control, CMS, CMS^TH−Gabbr1^ and CMS^TH−Gabbr1^-DBS groups. (K) The 24 h sucrose preference ratio, travel distance and center exploration time were tested in the Control, CMS, CMS^TH−Gabbr1^ and CMS^TH−Gabbr1^-DBS groups. All data are expressed as mean ± SEM. Multiple *t-test* analysis for (B)–(D), n = 4. *∗p* < 0.05, *∗∗p* < 0.01, CMS *vs*. Control, *∗p* < 0.05, *∗∗∗p* < 0.001, CMS^TH−Gabbr1^*vs*. CMS, *∗∗p* < 0.01, *∗∗∗∗p* < 0.0001, CMS^TH−Gabbr1^*vs*. CMS^TH-mCherry^, *∗∗∗∗p* < 0.0001, *∗∗p* < 0.01, CMS^TH−Gabbr1^*vs*. Control, and (H)–(K), n = 5. *∗∗∗p* < 0.001, *∗p* < 0.05, CMS^TH−Gabbr1^*vs*. CMS, *∗p* < 0.05, CMS^TH−Gabbr1^-DBS *vs*. CMS. Unpaired *t-test* for (E), n = 4. *∗p* < 0.05, TH^Gabbr1^-mCherry vs. TH-mCherry.

### Glutamate activation is involved in correcting the dopaminergic activity in the VTA and potentially involved in GABA_B_ expression alteration during NAcLat-DBS

Given the reduced GABA_B1_ expression of DA neurons after long-term DBS, we then examined the activity of excitatory neuron and glutamatergic input to the VTA following DBS. The DBS effects were assessed by calcium image recording of fiber photometry. The NAcLat-DBS (130 Hz, 100 μA, 100 μs pulse width) was given to mice to measure the VTA-DA neurons, GABA neurons and CAMKII neurons activity (Fig. 5A). We observed a significant increasing of CAMKII and DA neurons calcium signals, but an obvious fall of GABA neurons in the VTA region following DBS. The results displayed that the activity of GABA neurons rapidly decline, but the DA and CAMKII neurons synchronously were activated, appearing a remarkable peak. And the dopamine neurons activity subsequently declines earlier than the CAMKII neurons (Fig. 5B). When the activity of glutamate calcium fluorescence reached the peak and flattened out, the dopamine appeared a decline, and then the activity of two types of neurons gradually recovered until to baseline (Fig. 5B). Furthermore, glutamate (Glu) release in the VTA was measured in mice by recording fluorescence dynamics of iGluSnFR, a glutamate sensor after DBS. The results suggested that the glutamatergic input to the VTA present a prominent enhance following NAcLat-DBS (Fig. 5C and D) and the excitatory neuronal activity (c-Fos expression) increased after continuous DBS (Fig. 5E). Research have determined that glutamate controlled the cell membrane expression of GABA_B_ system in cultured neurons [36]. Hence, the membrane expression of GABA_B_ system in the VTA was examined by Western blot analysis in the Control, CMS and CMS-DBS mice (Fig. 5F). The levels of both membrane GABA_B1_ subunit, GABA_B2_ subunit and G protein-coupled inwardly rectifying potassium channel 2 (GIRK2) were significantly decreased in the VTA of CMS-DBS mice compared with the CMS mice (Fig. 5G–I). The dephosphorylation of serine 783 (ser-783) on the GABA_B2_ subunit is linked to reduced surface expression of GABA_B1_. There was a significant reduction in phosphorylation of the GABA_B2_ subunit after lasting DBS (Fig. 5J). These data show that the inhibitory input decreases and increased glutamatergic input in the VTA potentially involved in recovering DA neuronal activity following DBS. Therefore, the NAcLat-DBS enhances glutamate input, and the increased glutamatergic input is involved in correcting the dopaminergic activity after DBS.

**Fig. 5:**
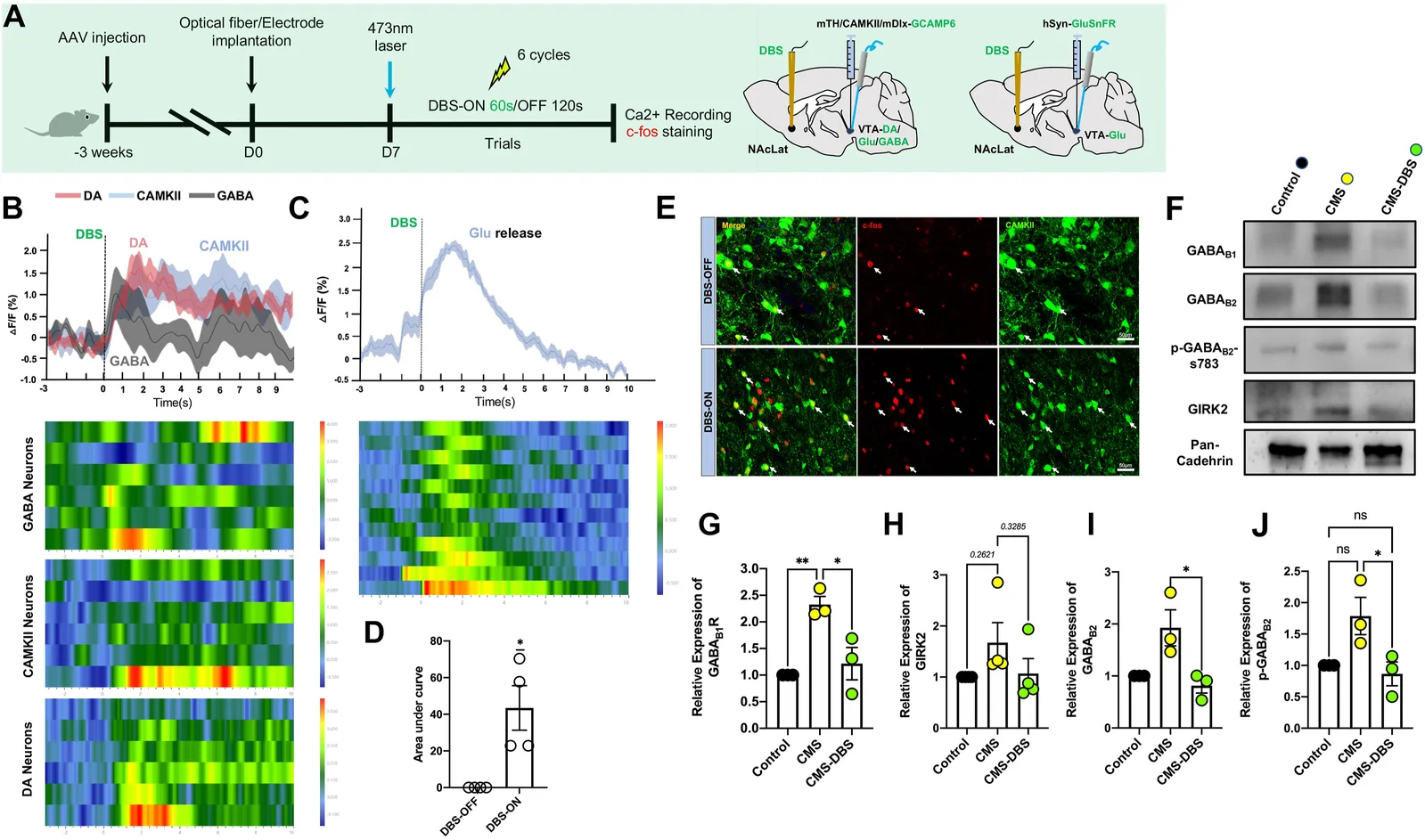
**Glutamate activation is involved in correcting the dopaminergic activity and potentially involved in GABA_B_ expression alteration during NAcLat-DBS** (A) Schematic of virus injection, optical fiber and electrode implantation and the experimental process of calcium image recording following DBS for 6 trials (one trial: 180s, DBS-ON 60s and DBS-OFF 120s, 6 trials). (B) Schematic of fiber photometry recordings of GABA, CAMKII and DA neurons calcium activity and the heatmap of GABA, CAMKII and DA neurons calcium activity after DBS. (C) Schematic of the glutamate neurotransmitter release task after DBS and the heatmap of glutamate sensor after DBS. (D) The area under curve about glutamate sensor after DBS-OFF and ON. (E) The c-Fos expression of CAMKII neurons in the VTA after DBS-ON compared with DBS-OFF. Green: CAMKII, Red: c-Fos, scale bar = 50 μm. (F) Schematic of the Western blot assay about GABA_B1_ system and the membrane protein GABA_B1_, GABA_B2_, *p*-GABA_B2_-s783 and GIRK2 expression in immunoblot assay in the Control, CMS and CMS-DBS mice. (G)–(J) The relative expression of membrane protein GABA_B1_, GABA_B2_, *p*-GABA_B2_-s783 and GIRK2 in the VTA. All data are expressed as mean ± SEM. Unpaired *t-test* analysis for (D), *∗p* < 0.05, DBS-ON *vs*. DBS-OFF, and Multiple *t-test* analysis for (G)–(J), n = 7. *∗∗p* < 0.01, *p* = 0.2621, CMS *vs*. Control, *∗p* < 0.05, *p* = 0.3285, CMS-DBS *vs*. CMS.

## Discussion

This study firstly demonstrates that NAcLat-DBS reduced DA GABA_B1_ expression in CMS mice to correct depressive behaviors. Furthermore, lasting DBS enhanced glutamatergic input to recover dopaminergic activity within the VTA. First, increased DA GABA_B1_ expression was observed in CMS mice, which was reversed by NAcLat-DBS, leading to increased DA release. Our study further shows that the NAcLat-DBS rescued the GABA_B_-agonist baclofen's ability to induce depressive symptoms that results from excessive excitement of GABA_B_. Importantly, overexpressing DA GABA_B1_ mainly in VTA-NAcLat circuit reversed the antidepressant effects of DBS in CMS mice, and the enhanced glutamate neurotransmitter involved in recovering dopaminergic activity in the VTA and potentially involved in GABA_B_ expression alteration during NAcLat-DBS. Together, these findings suggest a mechanism involving continuous DBS alleviating depression through which mainly correcting DA GABA_B_ expression exert beneficial clinical effects in MDD or TRD.

### Excessive increased DA GABA_B1_ expression in CMS mice

According to the results of GABA_B1_ immunohistochemistry, immunoblot and pharmacology manipulation, the increased DA GABA_B1_ expression and reduced dopamine activity in CMS mice were closely observed. These results indeed suggested that the increased DA GABA_B1_ expression enhanced the inhibitory effect on dopamine neurons [37,38]. GABA_B_ has been documented to exhibit strong expression in dopamine neurons across various midbrain regions, with a higher sensitivity to GABA_B_ receptors observed in dopamine neurons compared to GABA neurons in the VTA [13,39]. In addition, the strengthened GABA_B1_ expression will also influence the excitatory activity of dopamine neurons in CMS model mice.

The finding that increased GABA_B1_ expression of VTA-DA neurons contributes to despair and anhedonia behaviors in CMS mice (Fig. 4 and Supplementary Fig. 3) suggests DA GABA_B1_ expression variation potentially plays an important role in the hypodopaminergic pathogenesis of depressive-like behaviors [40]. Some preclinical and clinical data also determine that alterations in GABA_B_ receptor expression and function are associated with symptoms of major depressive disorder and response to antidepressant [[41], [42], [43], [44]]. Both the increased DA GABA_B1_ expression in the CMS mice model and reduced GABA_B1_R function in the alcohol addiction suggest the commonality of the dopamine excitement-inhibition imbalance mechanism in the psychiatry disease models. Moreover, animal studies have shown that long-term antidepressant use alters the GABA_B_ system [45,46], causing long-term increases in GABA_B_ neurotransmission rather than GABA_A_. Thus, the disruption of GABA_B_ system is a critical pathogenesis in psychiatric disorder related to reduced dopamine function, such as CMS mice, and a potentially key treatment target for MDD [41]. It has been documented that hypodopaminergic activity in the mesolimbic reward circuit exhibit considerable structural and functional remodeling following the pathological process of CMS [47]. In this context, enhanced DA GABA_B1_ expression in the VTA-NAcLat circuit could be considered one of the biological markers of impaired DA function and early pathological features of the animal model of CMS. Moreover, the modification of DA GABA_B1_ expression in the VTA-NAc reward circuit provides a reference point for intervention in the management of CMS.

### The downregulated DA GABA_B1_ expression mediated the activation effect of NAcLat-DBS to dopamine neurons in CMS mice

A chemical imbalance theory in part was the source of widespread use of pharmaco-therapies that produced brain-wide alterations in monoamine neurotransmitters such as dopamine in psychiatric diseases [48]. DBS constitutes a highly efficacious neuro-modulatory treatment approach [[49], [50], [51]]. NAc-DBS significantly influences the dopamine system and further research indicates that NAc shell (NAcS)-DBS elevates dopamine levels in the NAc and increases impulsive behaviors in mice [52,53]. Moreover, NAcS has been regarded as a central target for high-frequency stimulations (HFS) in mental disorders [54]. Based on the several cited studies [[55], [56], [57]], NAcLat-DBS was applied in the chronic mild stress-induced depression in the current study. While there is growing evidence of DBS's impact on the dopaminergic system, the precise molecular mechanism or target of NAcLat-DBS activating dopamine in the VTA-NAcLat circuit currently remains unknown.

In PD or essential tremor, DBS therapy mostly focused on emphasizing its beneficial response to local and network-wide stimulation, modulation of oscillatory activity [[58], [59], [60]], generating immediate therapeutic effects. But, DBS for severe or refractory depression (MDD and TRD) and other mental disorders usually requires long-term or prolonged intervention to achieve the therapeutic effect of anti-depression or symptoms improvement in clinical practice [23,61]. The significant finding in this article is that long-term NAcLat-DBS induced down-regulation of DA GABA_B1_ in the mesolimbic dopamine system, and antagonized depressive-like behaviors induced by baclofen, while main overexpression of DA GABA_B1_ in the VTA-NAcLat circuit reversed the effect of DBS in CMS mice. This work considers that the membrane expression of the G protein-coupled receptor GABA_B1_ mediated the antidepressant effects and regulation of DA neuronal activity by NAcLat-DBS.

#### Recovery of dopaminergic system and reduced DA GABA_B1_ expression are the reasons of NAcLat-DBS for improving despair and anhedonia

One of the key observations in current study is that NAcLat-DBS effectively activated the DA neuronal activity in VTA-NAcLat circuit. Additionally, dopamine signal plays a role in regulating reward and motivational arousal, and responsiveness to conditioned incentive stimuli [62,63]. Anhedonia in MDD has been linked to dysfunctions in the reward system, and in particular the DA system [64]. Neuroimaging, pharmacological and electrophysiological research in humans and animal models of depression have provided support for the presence of DA dysfunctions [65]. Previous studies have shown that the combination of fluoxetine with olanzapine, another atypical antipsychotic, induces an increase in DA, 5-HT, and norepinephrine extracellular levels in the prefrontal cortex [66,67]. In the VTA-NAcLat circuit, NAc presynaptic dopamine neurons receive synaptic projections from local and upstream cells to regulate their functional activities. And that NAcLat-DBS restores dopamine activity in CMS mice based on this circuit.

More recently, growing evidence suggests that GABA_B_ receptors are important target in the treatment of psychiatric disorders such as MDD [68]. Increasing emphasis has been placed on GABA_B1_ antagonism as a potential therapeutic strategy for depression [44,69]. Importantly, GABA_B_ is richly expressed in the midbrain dopamine system [70]. However, thus far, there is few evidence to demonstrate that GABA_B_ involve in modulating the VTA-DA neuronal activity in CMS mice. In this work, it was found that in vivo inhibiting or interfering DA GABA_B1_ expression in the VTA remarkedly alleviates CMS mouse's depressive-like behaviors, but activation or overexpression GABA_B1_ in DA neurons increased depression susceptibility in normal mouse. Therefore, the decreased dopamine activity and depressive-like phenotype in CMS mice is closely associated with upregulating expression of GABA_B1_. As previously noted, NAc-HFS has been shown to induce a hypoactivation of NAc local neural activities through GABA_B_ receptors [71]. Consistent with the previous studies, NAcLat-DBS increased dopamine neurotransmitter release into presynaptic terminal in the VTA and simultaneously decrease the expression of DA GABA_B1_ in this work. These finding confirmed the significant impact of GABA_B_ on dopamine activity in the depressive state, and also further demonstrated the importances of NAcLat-DBS correcting DA GABA_B1_ in antidepression and improving dopamine activity underlying the mesolimbic system. Taken together, the expression of DA GABA_B1_ is an important reason for CMS to produce hypodopaminergic activity. Our data add to current explanations regarding the potential causes of NAcLat-DBS correcting despair and anhedonia behaviors in depressive disorders.

#### The glutamatergic activation within the VTA induced by DBS involved in recovering dopaminergic activity, further potentially involved in GABA_B_ expression alteration

It should be pointed out that NAcLat-DBS induced an obvious Ca^2+^ fall signal of GABA neurons and a markedly Ca^2+^ elevation of glutamatergic neurons in the VTA region (Fig. 5). Researchers indicated that NAc-HFS mainly affects GABAergic medium spiny neurons (MSNs) and causes a subsequent increase of GABA outflow in NAc [72]. Using optogenetic stimulation have suggested that NAc inputs mainly target VTA GABA cells, rather than dopamine neurons [73,74]. It was reported that the influence of strengthened glutamatergic input on VTA-DA neurons is more relevant to its promoting effect on VTA-DA neuron excitation [75,76]. Therefore, it is that NAcLat-DBS increased the excitation of VTA-glutamate neurons in CMS mice through a process of disinhibition of glutamate neurons. Although VTA-GABA neurons are crucial for dopamine neuron disinhibition, increased glutamatergic inputs or/and glutamate neuronal activity in the VTA play a dominant role on membrane GABA_B_ expression [77] and act as another critical hub that drives the dopamine neuron excitation.

Previous research has found that the DA neurons disinhibition in the VTA contributed to the antidepressant effects targeting GABA_A_ during NAc-DBS [31]. Importantly, disruption of glutamate-excitatory projection neurons in the VTA that are responsible for circuit level information transmission could contribute to the GABA_B_ system membrane protein expression, and increased glutamate signaling may trigger GABA_B_ internalization. This speculation is grounded in existing literature on the crosstalk between glutamatergic activity and GABA_B_ receptor trafficking [77,78]. This work has been found that NAcLat-DBS targeted decreased the expression of DA GABA_B1_ to correct the depressive symptoms and improve the DA activity in CMS mice (Fig. 1, Fig. 2). This aids our understanding of the underlying mechanism behind DBS. In pyramidal neurons, the glutamate administration promoted GABA_B1_ to dynamically endocytose from membrane to cytoplasm, and decreased its availability on the membrane [40,[79], [80], [81]]. Furthermore, a TMS–EEG study confirmed decreased cortical inhibition originating from altered GABA_B1_ splicing in the prefrontal cortex of chronic alcoholics [82]. Further, we predicted that surface expression of GABA_B_ depending on glutamatergic input was necessary to the mechanism of NAcLat-DBS decreasing DA GABA_B1_ expression in present work. Importantly, although direct mechanistic verification is not achieved in the current study, our findings point out a correlational link between increased glutamate release and altered GABA_B_ expression. These observations deliver valuable foundational information and directional clues for in-depth mechanistic research (Fig. 5). Future studies will validate the causal linkage between increasing glutamate signaling and DA GABA_B1_ downregulation (targeted glutamatergic inhibition) and clarify the phosphorylated cascade mechanism of GABA_B_ endocytosis represent critical direction for our follow-up work.

The present study proposes that enhanced glutamatergic transmission recovered DA activity, further potentially involved in the internalization of GABA_B_. As an inevitable limitation of the current study, we cannot rule out other potential upstream factors (likely within astrocytes) that modulate GABA_B_ trafficking. Despite this shortcoming, our results demonstrate concurrent changes in increased glutamate levels and the surface expression of GABA_B_, providing correlational data and reasonable inferential clues for exploring GABA_B_ trafficking mechanism. In follow-up investigations, we will conduct pharmacological intervention experiments to further dissect the glutamate-excitatory mechanism of GABA_B_ internalization, and clarify how glutamatergic signaling regulates the trafficking process of GABA_B_ receptors. In addition, although our results revealed a consistent negative correlation between enhanced glutamatergic transmission and reduced DA GABA_B1_ expression under DBS intervention, whether these two molecular alterations, increased glutamate and reduced DA GABA_B1_, occur independently or through parallel compensatory pathways need to further determine for future mechanistic research.

## Conclusion and future perspectives

Our findings identify a previously unrecognized role of DA GABA_B1_ in mediating the antidepressant outcome of NAcLat-DBS and suggest a glutamate-excitatory mechanism involving alleviating dopaminergic activity and potentially correcting GABA_B_ expression to exert beneficial effects for treatment-resistant depression during NAcLat-DBS.

Several limitations need to be noted regarding the present study. First, the finding of increased DA GABA_B1_ expression in CMS mice in our study need to be validated in more depression models. Because CMS model is based on the dopamine function absence, it is not possible to expect the membrane GABA_B_ expression variation to fit other depression models. Subsequent verification with multiple independent depression models (e.g., chronic restraint stress, learned helplessness model) is required to confirm the specific correlation between DA GABA_B1_ expression alternation and depressive pathogenesis. Second, depression shows prominent gender disparities in clinical incidence and therapeutic responses. Considering sex-specific differences in depression pathophysiology and GABA_B_ receptor modulation, female subjects may exhibit distinct behavioral and molecular responses. Our experiments were performed in male mice, and these findings cannot be directly generalized to females. Besides, as an inherent limitation of in vivo electrical manipulation (DBS), slight local current diffusion to peripheral tissues cannot be completely eliminated. However, consistent anatomical localization across all subjects demonstrates that our experimental effects originate predominantly from NAcLat modulation. Further quantitative assessment of current spread will be pursued in follow-up work to better define the regional specificity of our results. Collectively, the current work will provide fresh perspectives on the molecular mechanism of DBS and etiology of TRD, thereby enabling the identification of potential targets for intervention.

## Author contributions

Jia-Ni Jing and Chao Yuan initiated and designed the research. Jia-Ni Jing wrote the manuscript. Jia-Ni Jing and Xia Li performed all experiments, Jia-Ni Jing and Hao Fan analyzed and interpreted the results. All authors approved the submission. We thank Academician Yi-Zheng Wang and his team from the Center of Military Brain Science, Institute of Military Cognition and Brian Sciences, Academy of Military Medical Sciences for their support of the experiment. We thank the staff from the Center of Military Brain Science animal facilities for their technical support and the members of the Physiopathology of post-traumatic stress disorder team for helpful discussions.

## Data availability

All data in this study are included in the article. Additional datasets generated and analyzed during the current study are available from the corresponding author upon reasonable request.

## Funding

The study was supported by grants from the 10.13039/501100001809National Natural Science Foundation of China (No. 32100954 and No. 3267090218).

## Declaration of interest statements

The authors declare that there is no conflict of interest relevant to the research described in this manuscript. All authors confirm that no financial support, commercial affiliations, funding relationships, or other potential conflicts of interest exist that could be perceived to bias the work, data interpretation, experimental design, results, or conclusions presented in this study. The research was conducted independently without any commercial or financial incentives that might compromise the objectivity, integrity, and scientific rigor of the work. All authors approve the final version of the manuscript and agree to be accountable for all aspects of the research.

## Figures and Tables

**figs1:**
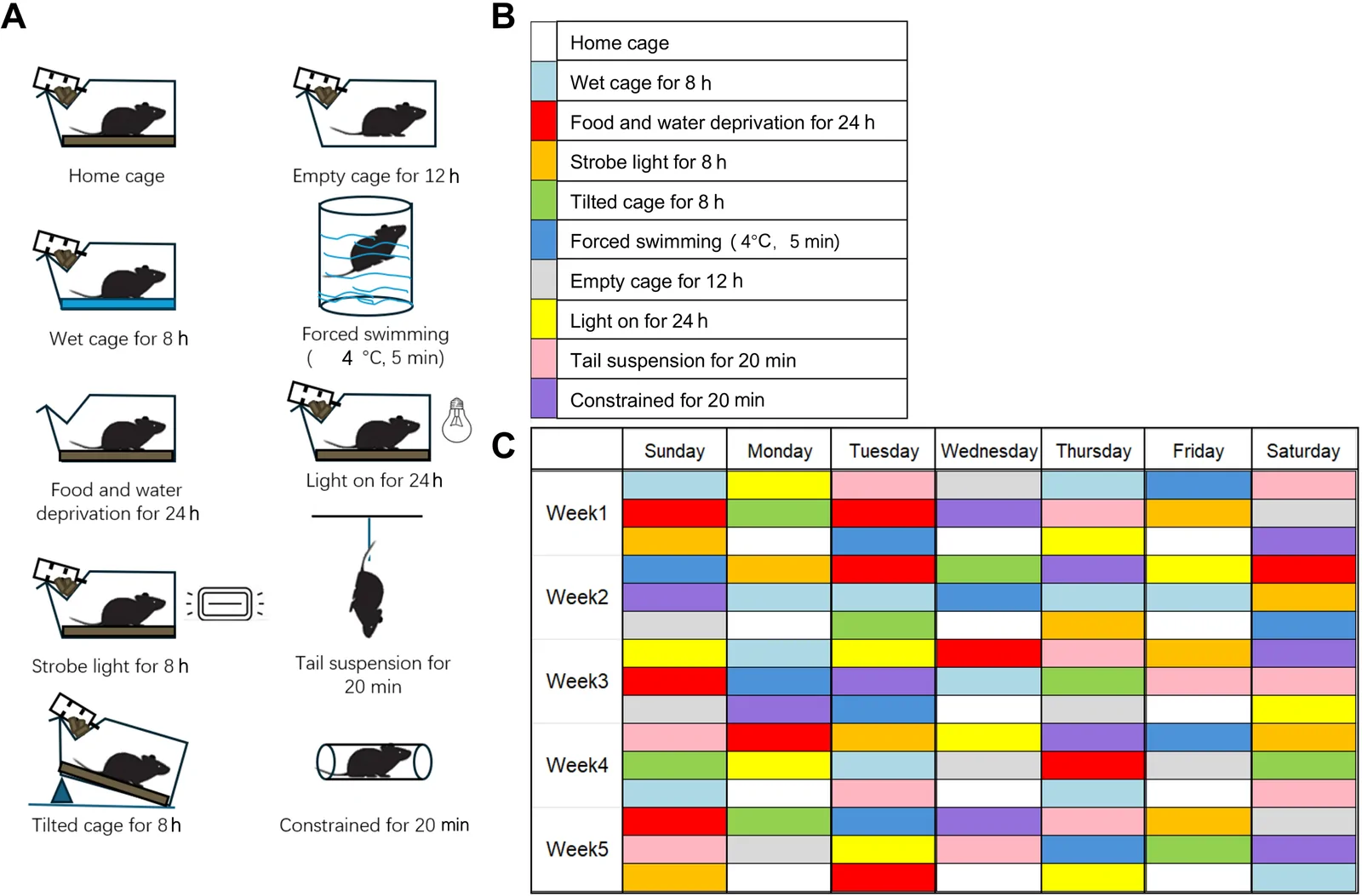


**figs2:**
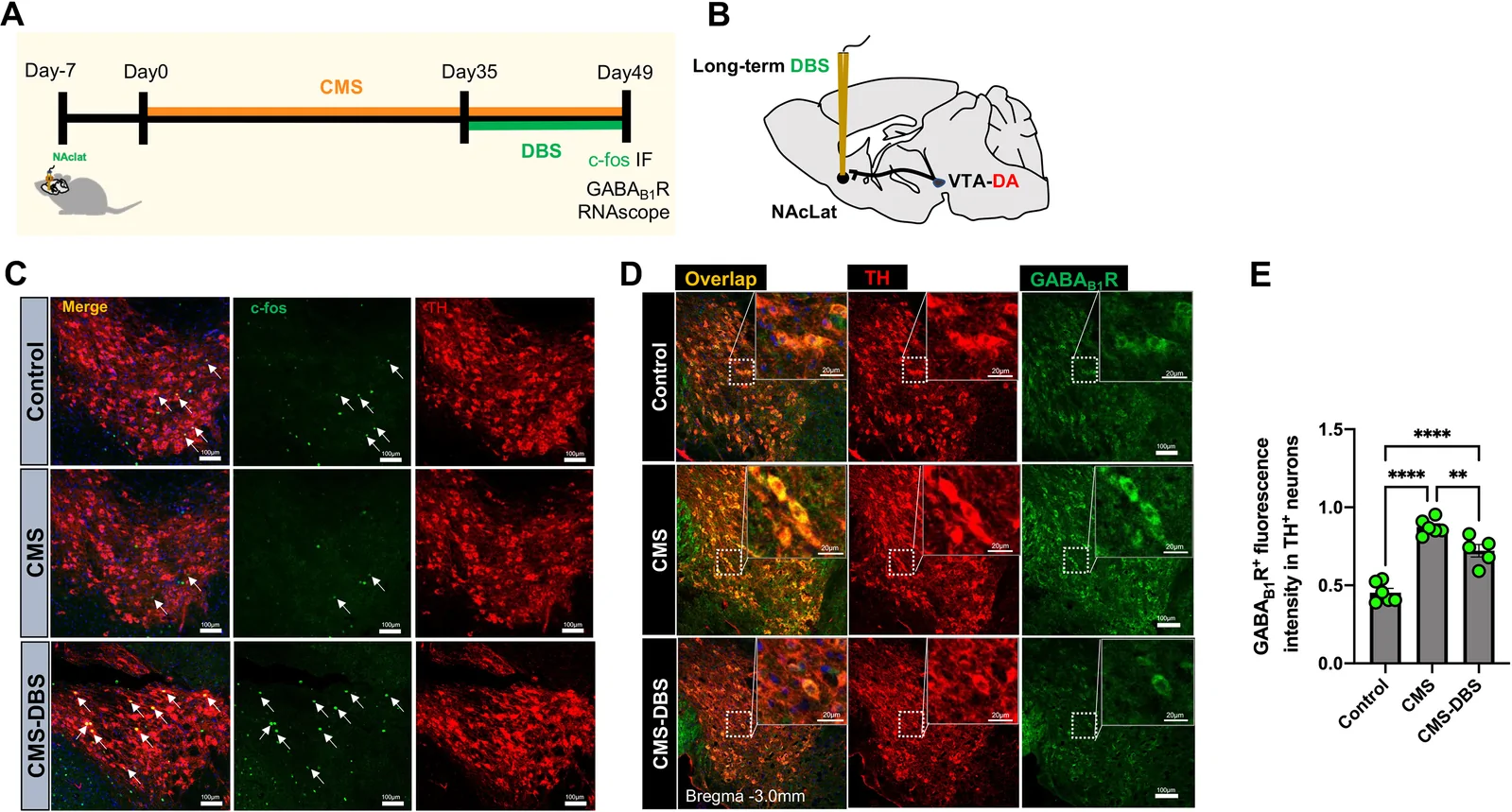


**figs3:**
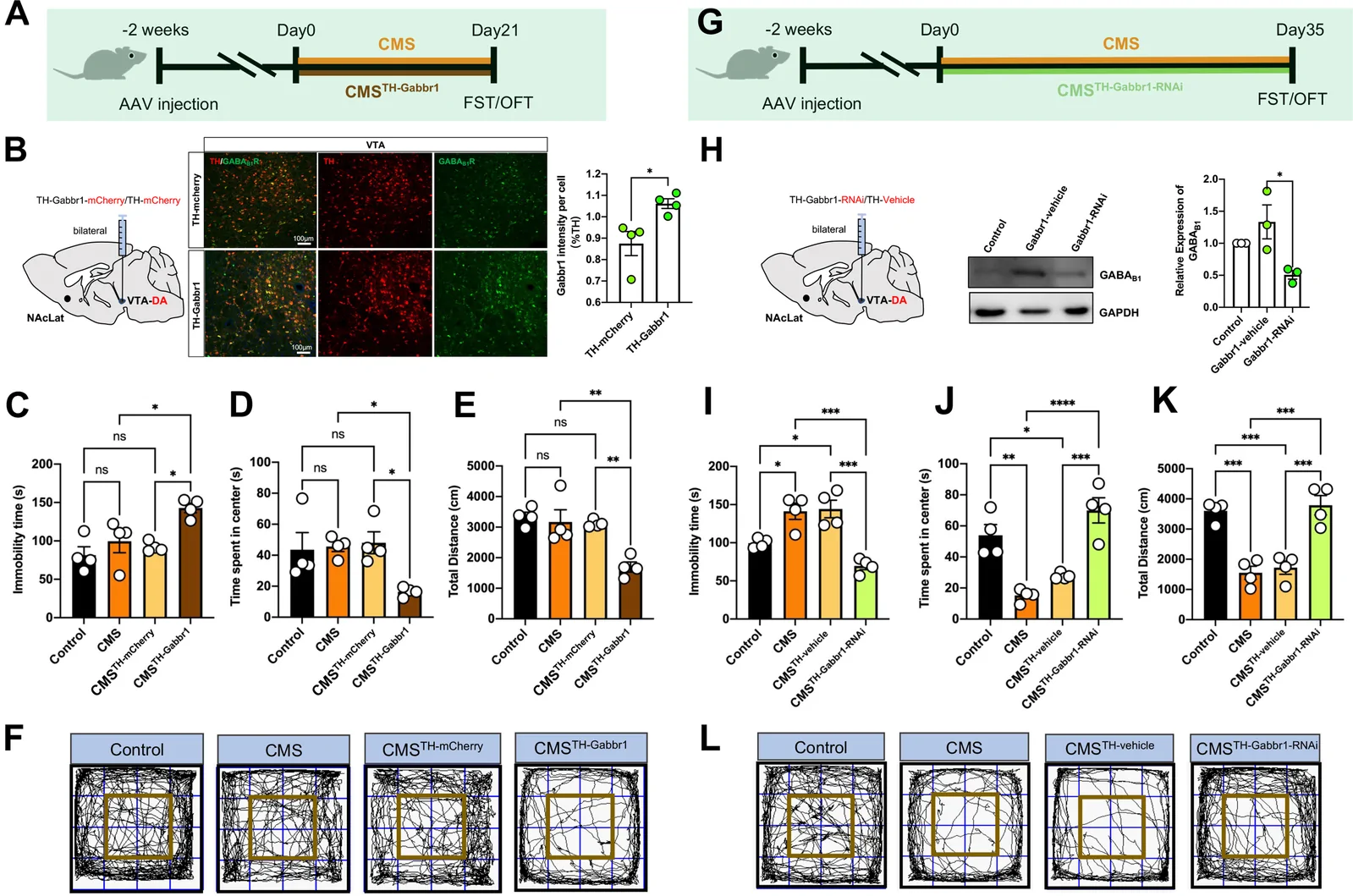


**figs4:**