# Supplementary material for: NAcLat-DBS reduces GABAB expression of dopamine neurons to correct depressive symptoms in CMS mice
Source: Neurotherapeutics. 2026 Aug 7;23(4):e01039. doi: 10.1016/j.neurot.2026.e01039 (PMC13639631; doi:10.1016/j.neurot.2026.e01039)
Supplement: Supplementary file 1 [file mmc1.docx]

**Supplementary Figure 1.** **Schematic overview of CMS protocol**

(A) Illustrations of different stressor procedures applied in CMS mouse model. (B) Color-coded schedule of stressors administered to mice over a five-week period and each color corresponds to a specific stressor, as indicated in the key. (C) Table summarizes the time and frequency of each stressor across days of the week.

**Supplementary Figure 2.** **NAcLat-DBS decreased DA GABA_B1_ expression in CMS mouse model**

(A) Experimental process of long-term DBS intervention in CMS mouse model. CMS mice were established during five weeks, the VTA-DA c-Fos and GABA_B1_ staining was tested after two weeks’ DBS (at 130 Hz). (B) Mice were bilaterally implanted with DBS electrode in the NAcLat. (C) The DA c-Fos expression of brain slices immunostaining in the Control, CMS and CMS-DBS groups (scale bar=100 μm). (D) Brain slices immunostaining was carried out after long-term DBS treatment in immunofluorescence (IF) for GABA_B1_ expression in VTA DA neurons (scale bar=100 μm, scale bar=20 μm in the white box). (E) The fluorescence intensity of GABA_B1_R^+^ cell in dopamine neurons in Control, CMS and CMS-DBS mice. All data are expressed as mean ± SEM. Multiple *t-test* analysis for (E), n=5, *****p*<0.0001, CMS *vs*. Control, CMS-DBS *vs*. Control, ***p*<0.01, CMS-DBS *vs.* CMS.

**Supplementary Figure 3.** **Transfecting GABA_B1_ in VTA dopamine neurons augment depression susceptibility during chronic mild stress**

(A) Experimental process of the virus injection (AAV_9_-TH-Gabbr1/AAV_9_-TH-mCherry) and chronic mild stress modeling. (B) Left: the virus injection of AAV_9_-TH-mCherry/AAV_9_-TH-Gabbr1-mCherry in the VTA; Right: Gabbr1 intensity in the DA neurons of TH-mCherry and TH-Gabbr1 groups images of overexpression GABA_B1_ in DA neurons. Red: TH; Green: GABA_B1_. Scale bars=100 μm. (C) The force swim immobility time in the Control, CMS, CMS^TH-mCherry^ and CMS^TH-Gabbr1^ groups subjecting 21 days of CMS. (D)-(E) The time spent in center and total distance were tested in the Control, CMS, CMS^TH-mCherry^ and CMS^TH-Gabbr1^ groups subjecting 21 days of CMS. (F) Track diagram of spontaneous activity in the Control, CMS, CMS^TH-mCherry^ and CMS^TH-Gabbr1^ groups. (G) Experimental process of the virus injection (AAV_9_-Gabbr1-RNAi/AAV_9_-TH-vehicle) and chronic mild stress modeling. (H) Left: the virus injection of AAV_9_-TH-vehicle/ AAV_9_-Gabbr1-RNAi in the VTA; Right: the representative GABA_B1_ expression in the VTA. (I) The force swim immobility time in the Control, CMS, CMS^TH-vehicle^ and CMS^TH-Gabbr1-RNAi^ groups subjecting 35 days of CMS. (J)-(K) The time spent in center and total distance were tested in the Control, CMS, CMS^TH-vehicle^ and CMS^TH-Gabbr1-RNAi^ groups subjecting 35 days of CMS. (L) Track diagram of spontaneous activity in the Control, CMS, CMS^TH-vehicle^ and CMS^TH-Gabbr1-RNAi^ groups. All data are expressed as mean ± SEM. Multiple *t-test* analysis for (C)-(E), n=4. **p*<0.05, ***p*<0.01, CMS^TH-Gabbr1^ *vs*. CMS^TH-mCherry^, **p*<0.05, ***p*<0.01, CMS^TH-Gabbr1^*vs*. CMS, and (I)-(K), n=4. **p*<0.05, ***p*<0.01, ***p<0.001, CMS *vs*. Control, **p*<0.05, ****p*<0.001, CMS^TH-vehicle^ *vs*. Control，****p*<0.001, CMS^TH-Gabbr1-RNAi^ *vs*. CMS^TH-vehicle^，****p*<0.001， *****p*<0.0001, CMS^TH-Gabbr1-RNAi^ *vs*. CMS. Unpaired *t-test* for (B), n=4. **p*<0.05, TH-Gabbr1 vs. TH-mCherry.

**Supplementary Figure 4. Inhibition of GABA_B_ increased CMS mice’s center exploration time in the open field test**

(A-B) Experimental scheme of the intraperitoneally CGP35348 (1 mg/kg/d) administration for fourteen days in the Control and CMS groups. (C) Track diagram of spontaneous activity after CGP35348 administration for 0 day, 3 days, 7 days, 10 days and 14 days in the OFT. (D) Line graph: the change of total distance during CGP administration in the Control and CMS groups. (E) Line graph: the time spent in the center during CGP administration in the Control and CMS groups. All data are expressed as mean ± SEM. Repeated-measures ANOVA followed by post-hoc tests analysis for (B) and (D), n=4. ***p*<0.01, ****p*<0.001, CMS *vs.* Control.
